# Methionine-driven YTHDF1 expression facilitates bladder cancer progression by attenuating RIG-I-modulated immune responses and enhancing the eIF5B-PD-L1 axis

**DOI:** 10.1038/s41418-024-01434-y

**Published:** 2024-12-13

**Authors:** Anze Yu, Liangmin Fu, Lanyu Jing, Yinghan Wang, Zifang Ma, Xinwei Zhou, Rui Yang, Jinhui Liu, Jiao Hu, Wei Feng, Taowei Yang, Zhenhua Chen, Xiongbing Zu, Wei Chen, Junxing Chen, Junhang Luo

**Affiliations:** 1 Department of Urology, First Affiliated Hospital, Sun Yat-sen University, Guangzhou, Guangdong China; 2 Department of Urology, The Second Xiangya Hospital, Central South University, Changsha, Hunan China; 3 Breast Department, Guangdong Provincial Hospital of Chinese Medicine, The Second Affiliated Hospital of Guangzhou University of Chinese Medicine, The Second Clinical College of Guangzhou University of Chinese Medicine, Guangdong Provincial Academy of Chinese Medical Sciences, Guangzhou, Guangdong China; 4 Department of Breast Surgery, First Affiliated Hospital, Sun Yat-sen University, Guangzhou, Guangdong China; 5 Department of Urology, Hengyang Central Hospital, Hengyang, Hunan China; 6 Department of Burns, Wound Repair and Reconstruction, First Affiliated Hospital of Sun Yat-sen University, Guangzhou, Guangdong China; 7 Department of Urology, Xiangya Hospital, Central South University, Changsha, Hunan China; 8 Department of Pancreato-Biliary Surgery, The First Affiliated Hospital of Sun Yat-sen University, Guangzhou, Guangdong China

**Keywords:** Cell death and immune response, Tumour biomarkers

## Abstract

The impact of amino acids on tumor immunotherapy is gradually being uncovered. In this study, we screened various essential and non-essential amino acids and found that methionine enhances mRNA methylation and reduced the activation of Type I interferon pathway in bladder cancer. Through RNA sequencing, point mutations, MB49 mouse tumor models, and single-cell RNA sequencing, we demonstrated that high methionine levels elevate the expression of m^6^A reader YTHDF1, promoting the degradation of RIG-I, thereby inhibiting the RIG-I/MAVS-mediated IFN-I pathway and reducing the efficacy of tumor immunotherapy. Additionally, immunoprecipitation and mass spectrometry revealed that YTHDF1 binds to the eukaryotic translation initiation factor eIF5B, which acts on PD-L1 mRNA to enhance its translation and promote immune evasion. By intravesical administration of oncolytic bacteria VNP20009, we effectively depleted methionine locally, significantly prolonging mouse survival and enhancing immune cell infiltration and differentiation within tumors. Multiplex immunofluorescence assays in bladder cancer immunotherapy patients confirmed our findings. Our research elucidates two mechanisms by which methionine inhibits bladder cancer immunotherapy and proposes a targeted methionine depletion strategy that advances research while minimizing nutritional impact on patients.

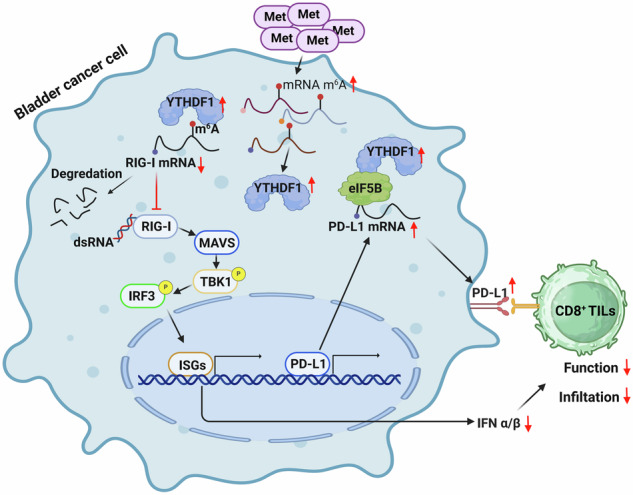

## Introduction

Bladder cancer (BC) stands as one of the most prevalent malignant diseases of the urinary system. The advent of immune checkpoint blockade (ICB) therapy, particularly anti-PD-1/PD-L1 treatment, has presented remarkable survival benefits in patients with advanced muscle-invasive bladder cancer (MIBC), fundamentally reshaping the treatment landscape [1–6]. Nonetheless, many patients still undergo unsatisfactory clinical outcomes [7–9]. A thorough comprehension of the fundamental cellular and molecular mechanisms underlying antitumor immunity may lead to optimized treatment strategies [10–13]. Previous research has suggested that the effectiveness of immunotherapy hinges on T cell-related immune responses [14]. The IFN-I signal proves pivotal for specific antitumor T cell responses, primarily dependent on the activation of pattern recognition receptors (PRRs) of the innate immune system [15–19]. The type I interferon pathway was previously deemed primarily existent in anti-infection immunity; however, recent studies have discovered that potent IFN-related immune responses can be induced by activating nucleic acid sensors in tumor cells through means such as chemotherapy, thus achieving antitumor immune responses, a phenomenon termed “viral mimicry” [16, 20–22]. These pathways also serve as core mechanisms for enhancing antitumor immune responses with various neoadjuvant chemotherapy drugs [14]. However, these pathways appear to be absent or weakened in various cancer types.

Nutrition significantly impacts health, with dietary interventions often utilized in treating metabolic diseases [23]. Research indicates that restricting the intake of certain essential amino acids can affect cancer progression and treatment outcomes [24]. Methionine, as an essential amino acid, exhibits significant variability in human plasma and plays critical roles in cellular functions, including methylation reactions, maintaining redox balance, and folate metabolism [25–28]. Recent studies have demonstrated the importance of methionine for the survival of both tumor cells and T cells, with T cells exhibiting higher sensitivity to methionine deficiency compared to tumor cells [27–30]. Additionally, studies have shown that dietary methionine restriction enhances the response to immune checkpoint inhibitors in a colorectal cancer model [31]. Tumor cells induce T cell apoptosis by depriving methionine and affecting one-carbon metabolism and methionine cycling [29]. However, it remains unclear whether reducing methionine intake through diet affects the efficacy of immune checkpoint blockade (ICB) therapy.

Recent studies have indicated that m^6^A levels are significantly elevated in bladder tumors compared to normal bladder tissues, with most research attributing this to the abnormal expression of m^6^A methyltransferases METTL3 and METTL14 [32–34]. These studies suggest that METTL3 and METTL14 enhance the stability of various oncogenic mRNAs, facilitating tumor cell self-renewal, invasion, metastasis, and overall tumor progression [32, 35, 36]. However, the precise role of m^6^A readers in the context of bladder cancer immunotherapy remains unclear. Our research demonstrates that methionine inhibition can reduce the expression of YTH domain family protein 1 (YTHDF1), an m^6^A reader primarily responsible for regulating mRNA stability. Current research indicates that tumor-intrinsic YTHDF1 promotes MHC-I degradation, leading to tumor immune evasion and inducing resistance to immune checkpoint inhibitors [37]. In this work, we confirm YTHDF1’s role in promoting mRNA translation and also uncover its potential to facilitate the degradation of certain mRNAs.

In this study, through screening essential and non-essential amino acids in bladder cancer cell culture media, we demonstrated that methionine metabolism regulates IFN-I production by modulating the RIG-I/MAVS pathway. Additionally, we showed that methionine metabolism regulates PD-L1 expression through the YTHDF1-eIF5B axis, thereby influencing the response to immunotherapy. We unveiled novel mechanistic connections among methionine metabolism, IFN-I signaling, m^6^A methylation, and antitumor immunity, and proposed that dietary intervention with methionine, local methionine deprivation in bladder tumors, or targeting the m^6^A-specific reader YTHDF1 may represent potential therapeutic approaches for antitumor immunotherapy.

## Materials and methods

A comprehensive description of the materials and methods can be found in the Online Supplementary Materials.

## Results

### Methionine alters antitumor immunity in bladder cancer via m^6^A methylation changes and type I interferon signaling

**Fig. 1:**
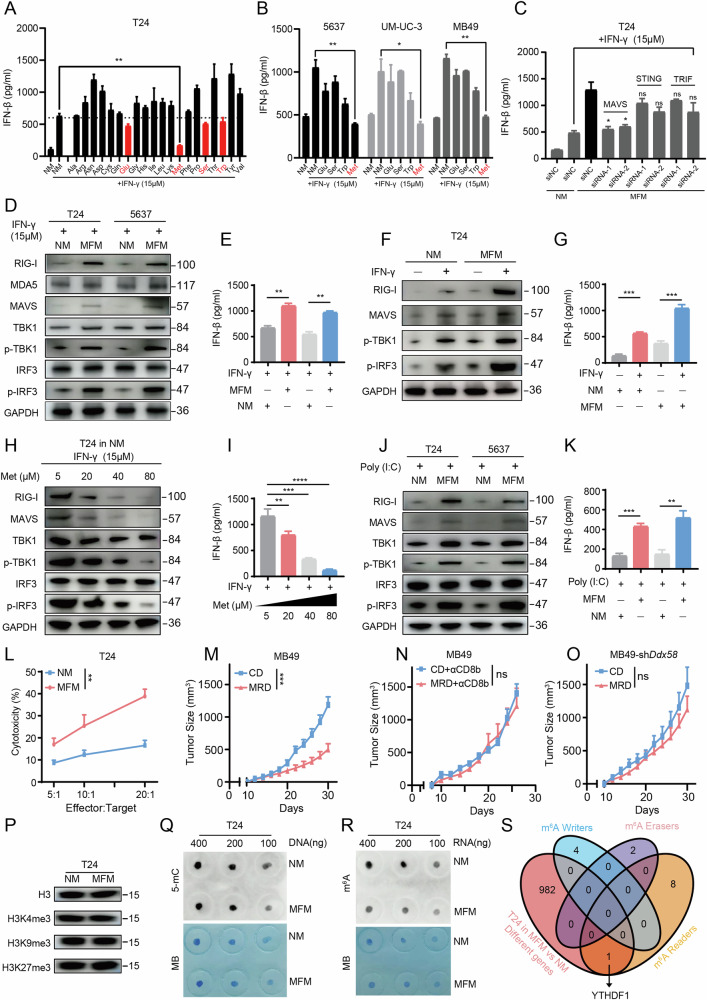
Methionine alters antitumor immunity in bladder cancer via m^6^A methylation changes and type I interferon signaling. **A** ELISA results showing the impact of adding 20 essential and non-essential amino acids to the culture medium on IFN-β release in T24 cells. **B** The effect of adding five specific amino acids to the culture medium on IFN-β release in 5637, UM-UC-3, and MB49 cell lines. **C** ELISA was used to measure IFN-β content in the supernatant after knockdown of specific genes and culturing in medium with or without methionine. Western Blot (**D**) and ELISA (**E**) detecting the expression of MAVS-related type I interferon pathway proteins and IFN-β content in the supernatant of T24 and 5637 cell lines after culturing in NM and MFM with IFN-γ treatment. Western Blot (**F**) and ELISA (**G**) detecting the expression of RIG-I-MAVS-related type I interferon pathway proteins and IFN-β content in the supernatant of T24 cells after culturing in NM and MFM with or without IFN-γ treatment. Western Blot (**H**) and ELISA (**I**) detecting the expression of RIG-I-MAVS-related type I interferon pathway proteins and IFN-β content in the supernatant of T24 cells after adding different concentrations of methionine. Western Blot (**J**) and ELISA (**K**) detecting the expression of RIG-I-MAVS -related type I interferon pathway proteins and IFN-β content in the supernatant of T24 cells after culturing in NM and MFM with poly (I:C) treatment. **L** LDH cytotoxicity assay detecting the percentage of cytotoxicity after co-culturing T24 cells with activated human PBMCs under NM and MFM conditions. **M** Tumor growth of MB49 in mice under CD and MRD feeding conditions. **N** Tumor growth of MB49 in mice under CD and MRD feeding conditions after administration of anti-CD8b antibody. **O** Tumor growth of MB49 in mice under CD and MRD feeding conditions after knocking down the Ddx58 gene in MB49 cells. **P** Western Blot detecting the expression levels of major histones and methylated histones in T24 cells under NM and MFM conditions. **Q** Dot Plot detecting the expression level of 5-mC in T24 cells under NM and MFM conditions. **R** Dot Plot detecting the expression level of m^6^A in T24 cells under NM and MFM conditions. **S** Venn diagram showing the intersection of differentially expressed genes and m^6^A-related molecules in T24 cells under NM and MFM conditions detected by RNA-seq. All p values are less than 0.05, indicating statistical significance. Error bars represent Mean ± SD. Three biologically independent experiments were performed. ns, P > 0.05, *P < 0.05, **P < 0.01, ***P < 0.001, ****P < 0.0001. NM normal Medium, MFM methionine free medium, CD complete diet, MRD methionine restricted diet.

Studies have reported dysregulation of amino acid metabolism in tumor cells [24, 38, 39]. To investigate whether this dysregulation affects type I interferon (IFN-I) production during immunotherapy, we treated T24 human bladder cancer cells with IFN-γ to simulate the immune microenvironment. We also supplemented the medium with specific amino acids. ELISA analysis showed that IFN-γ induced strong IFN-β expression, but IFN-β expression decreased in cells cultured with Glu, Met, Ser, or Trp. Methionine supplementation caused the most significant reduction (Fig. 1A). To verify the generality of this result in bladder cancer, we supplemented the culture medium of 5637, UMUC-3, and MB49 cells with the aforementioned amino acids. Consistent with previous observations, methionine supplementation significantly downregulated IFN-β expression in all three cell lines (Fig. 1B).

By screening adapters of three systems—STING, MAVS, and TRIF—using siRNA, we found that knocking down MAVS significantly inhibited IFN-γ-induced IFN-β expression, while interference with the other two adapters had no effect (Fig. 1C). Since MAVS can be activated by RIG-I (DDX58) and MDA5, we conducted Western blotting in T24 and 5637 cell lines to assess which sensor is affected by methionine. Results showed that RIG-I expression increased significantly in both cell lines cultured in methionine-free medium, while MDA5 showed little change. We then knocked out RIG-I in 5637 cells and measured IFN-β production under various conditions (with or without IFN-γ and methionine). Results indicated that under IFN-γ stimulation, RIG-I knockout cells maintained a certain level of IFN-β during methionine restriction, with secretion significantly higher than in the methionine-supplemented group, but still lower than in RIG-I wild-type controls. In the absence of IFN-γ, no significant differences in IFN-β production were observed in RIG-I knockout cells, regardless of methionine restriction (Fig. S1A). Additionally, MAVS, p-TBK1, and p-IRF3 expression increased (Fig. 1D). To explore whether methionine-induced IFN-β release is controlled by RIG-I or MAVS, we investigated the link between methionine metabolism and MAVS regulation, particularly the effects of m^6^A methylation. RNA stability assays showed that MAVS RNA was more stable in cells cultured in MFM medium compared to NM medium (Fig. S1B). Then, we performed RIP-qPCR assays with m^6^A and IgG antibodies to evaluate m^6^A -modified MAVS expression. Results showed that MAVS transcripts are methylated, with higher levels in NM (Fig. S1C), indicating methionine affects MAVS stability via m^6^A modification. Next, we knocked out RIG-I in 5637 cells and overexpressed either an empty vector or MAVS under IFN-γ stimulation and methionine restriction. ELISA revealed that MAVS overexpression in RIG-I knockout cells increased IFN-β secretion compared to empty vector controls, though levels remained below those in wild-type cells. This trend persisted under methionine-replete conditions, with overall lower IFN-β levels than under methionine restriction, suggesting methionine’s effects on MAVS are primarily mediated via RIG-I (Fig. S1D). ELISA of culture supernatants confirmed similar trends observed in western blot analyses (Fig. 1E). Further, western blot and ELISA of T24 cells treated with IFN-γ in NM or MFM revealed the most significant IFN-I pathway protein expression under IFN-γ and MFM conditions (Fig. 1F-G). Lastly, varying methionine concentrations in T24 cells showed a dose-dependent decrease in IFN-I pathway protein expression (Fig. 1H-I). Subsequently, we treated T24 and 5637 cell lines cultured in normal medium and methionine-free medium with the synthetic double-stranded RNA poly (I:C). Similarly, in both cell lines cultured in methionine-free medium, the expression levels of proteins such as RIG-I, MAVS, p-TBK1, and p-IRF3 were higher compared to the normal medium group (Fig. 1J-K). Additionally, to assess whether reduced methionine availability disrupts self-tolerance to endogenous RNA [40], we utilized the methionine synthase reductase 1 (MTR1) inhibitor TFMT (Trifluoromethyl-tubercidin). As illustrated in Fig. S1E, the addition of TFMT significantly elevated IFN-β levels in T24 cells under methionine-deprived conditions compared to the methionine-deprived group without TFMT, with this difference reaching statistical significance. The lowest IFN-β release was observed in the methionine-sufficient, no TFMT group, whereas the methionine-sufficient group with TFMT showed a moderate increase in IFN-β release. However, this increase did not reach the levels observed in the methionine-deprived + TFMT group. This pattern was corroborated in another cell line, 5637. These findings suggest that in our experimental model, methionine deprivation modestly weakens self-tolerance to endogenous RNA, triggering an IFN response likely through the presence of unmethylated endogenous RNA.

Next, we co-cultured T24 cells with activated human PBMCs in NM and MFM media and measured LDH release. The results indicated that the co-culture system in MFM media exhibited stronger cytotoxicity compared to that in NM media at different cell ratios (Fig. 1L). Subsequently, we inoculated C57BL/6 mice with MB49 bladder cancer cells and fed them either a complete diet (CD) or a methionine-restricted diet (MRD). The results demonstrated that tumor growth was significantly slower in the MRD group compared to the CD group (Fig. 1M). Additionally, after the injection of αCD8b antibody, the difference in tumor growth between the two groups disappeared, indicating that MRD primarily exerted its effects through CD8^+^ T cells (Fig. 1N). Furthermore, when MB49 tumor cells with knocked-down Ddx58 were implanted into mice from both the CD and MRD groups, there was no significant difference in tumor growth between the two groups, suggesting that the inhibitory effect of MRD on tumor growth requires the involvement of IFN-I (Fig. 1O). To distinguish between the downstream effects of type I IFN signaling and the direct consequences of methionine deprivation, we knocked down IFNAR1 in T24 and 5637 cells and measured IFN-β release using ELISA under conditions of IFN-γ stimulation, both with and without methionine. The results indicated that IFNAR1 knockdown had no effect on IFN-β release in either cell line (Fig. S2A). Next, we co-cultured T24-shNC and T24-shIFNAR1 cells with activated human CD8^+^ T cells in normal medium (NM) or methionine-free medium (MFM) and performed an LDH assay to measure cytotoxicity. The results showed that the T24-shNC + MFM group exhibited the highest tumor-killing effect, followed by the T24-shNC + NM and T24-shIFNAR1 + MFM groups. The T24-shIFNAR1 + NM group displayed the weakest killing effect (Fig. S2B). At the animal level, we knocked down IFNAR1 in MB49 cells and subcutaneously implanted MB49-shNC and MB49-shIFNAR1 cells into C57BL/6 mice under complete diet (CD) or methionine-restricted diets (MRD). The results showed that tumor growth was fastest in the MB49-shIFNAR1 + CD group, with the fewest CD8^+^ T cells infiltrating per milligram of tumor tissue. Tumor growth in the MB49-shNC + CD and MB49-shIFNAR1 + MRD groups was slower, with more CD8^+^ T cell infiltration. The MB49-shNC + MRD group had the slowest tumor growth, accompanied by the highest CD8^+^ T cell infiltration (Fig. S2C–F). Additionally, the proportion of TNF-α^+^ IFN-γ^+^ CD8^+^ tumor-infiltrating lymphocytes (TILs) among all CD8^+^ TILs was lowest in the MB49-shIFNAR1 + CD group, followed by the MB49-shNC + CD and MB49-shIFNAR1 + MRD groups, with the highest proportion observed in the MB49-shNC + MRD group (Fig. S2G).

Given the crucial physiological role of methionine in providing methyl groups, it is involved in common methylation processes, including histone methylation, 5-mC DNA modification, and m^6^A RNA modification. Therefore, we conducted assays for histone (Fig. 1P), 5-mC (Fig. 1Q), and m^6^A (Fig. 1R) modifications, with m^6^A methylation showing the most pronounced changes. To elucidate which specific m^6^A-related proteins serve as key downstream regulators of methionine, we cultured T24 cells in both NM and MFM media, followed by RNA sequencing and intersection analysis of differential genes with m^6^A-related molecules. Interestingly, we found that only the expression of YTHDF1 intersected with the aforementioned differential genes (Fig. 1S).

### YTHDF1 is associated with poor immune infiltration and prognosis and is negatively correlated with RIG-I expression in bladder cancer

To elucidate how methionine regulates YTHDF1 expression, we conducted an RNA degradation assay and found that YTHDF1 RNA degradation was slower in normal medium compared to methionine-free medium in both T24 and 5637 cells, with statistically significant differences. This suggests that methionine helps maintain YTHDF1 RNA stability (Fig. S3A). Additionally, in the methionine cycle, methionine is converted into its active form, SAM, in the presence of ATP. SAM donates methyl groups for various physiological processes and is subsequently converted into SAH, which is then hydrolyzed to homocysteine (Fig. S3B). We hypothesized that methylation enhances YTHDF1 mRNA stability. To test this, T24 and 5637 cells were treated with methionine, SAM, SAH, and homocysteine. We observed an increase in YTHDF1 RNA and protein expression with methionine and SAM treatment, but a decrease with SAH and homocysteine, likely due to competition for methyl groups ((Fig. S3C-D).

**Fig. 2:**
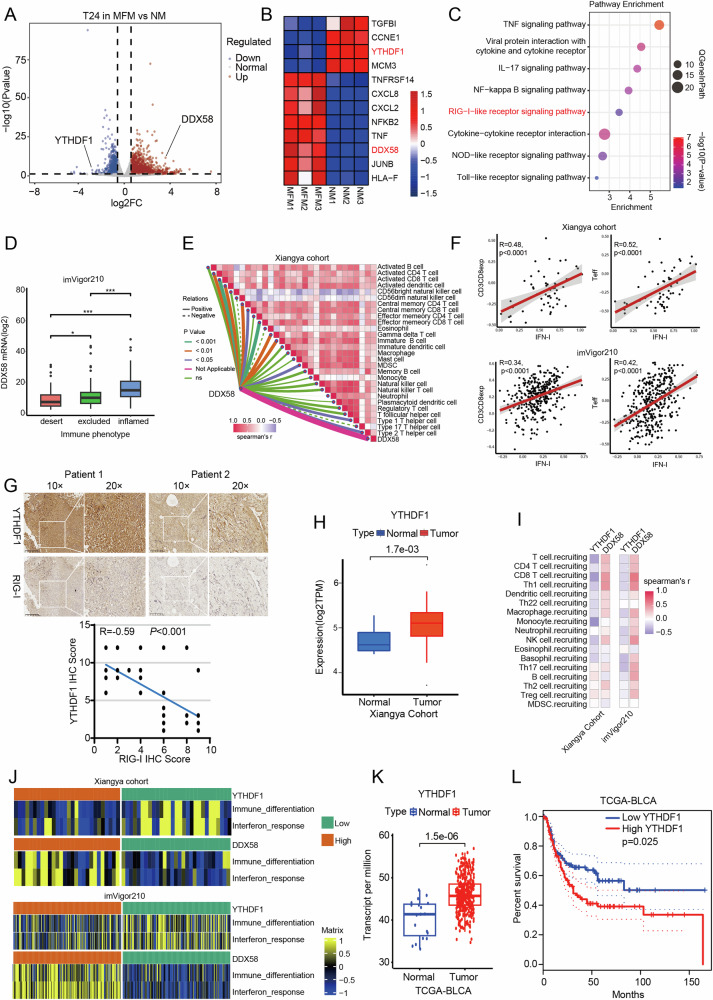
YTHDF1 is associated with poor immune infiltration and prognosis and is negatively correlated with RIG-I expression in bladder cancer. Volcano plot (**A**) and heatmap (**B**) showing differential gene expression in T24 cells under NM and MFM culture conditions. **C** Enrichment pathways of differentially expressed genes. **D** Correlation between DDX58 and bladder cancer immune infiltration subtypes in the imVigor210 cohort. **E** The correlation between DDX58 expression and immune cell infiltration in Xiangya cohort. **F** Correlation between type I interferon expression and CD8^+^ T cell infiltration and effector T cells in Xiangya cohort and the imVigor210 cohort. **G** IHC showing the correlation between YTHDF1 and RIG-I expression in bladder cancer patients from Xiangya cohort. **H** Differences in YTHDF1 expression between tumor tissues and adjacent normal tissues in Xiangya cohort. **I** Correlation between YTHDF1 and DDX58 with immune cell recruitment in Xiangya cohort and the imVigor210 cohort. **J** Correlation between YTHDF1 and DDX58 with immune-related pathways in Xiangya cohort and the imVigor210 cohort. **K** YTHDF1 expression differences between tumor tissues and adjacent normal tissues in the TCGA-BLCA cohort. **L** Correlation between YTHDF1 expression and prognosis in the TCGA-BLCA cohort. *P < 0.05, ***P < 0.001. NM normal medium, MFM methionine free medium.

In the RNA sequencing results mentioned above, we found that methionine might regulate the IFN-I pathway by modulating the m^6^A reader YTHDF1. Interestingly, the sequencing results also revealed a negative correlation between the expression of DDX58 and YTHDF1 (Fig. 2A). Concurrently, DDX58 expression exhibited a similar trend with the expression of various immune infiltration or cytokine genes, such as CXCL8 and TNF (Fig. 2B). The enrichment analysis of the sequencing results indicated that the differentially expressed genes were enriched in the RIG-I-like receptor signaling pathway (Fig. 2C). These results suggest that methionine may influence the release of IFN-I by negatively regulating DDX58 through YTHDF1. Subsequently, we examined the expression of DDX58 in the imVigor210 bladder cancer immunotherapy cohort and found that DDX58 expression increased with the degree of immune infiltration (Fig. 2D). To further explore the relationship between YTHDF1, DDX58, and IFN molecules, we performed analyses across three cohorts. Firstly, in the Xiangya cohort of bladder cancer patients established at our center, we found that DDX58 expression was positively correlated with the recruitment and infiltration of various immune cells (Fig. 2E). Additionally, IFN-I exhibited a strong positive correlation with CD8^+^ T cells and effector T cells (Fig. 2F, upper). Immunohistochemical analysis of bladder cancer tissues further confirmed a negative correlation between YTHDF1 and DDX58 expression in tumors (Fig. 2G). The Xiangya cohort also validated that YTHDF1 expression was higher in tumor tissues compared to adjacent normal tissues (Fig. 2H). Furthermore, YTHDF1 expression was negatively correlated with immune cell recruitment (Fig. 2I, left) and the activation of immune-related pathways (Fig. 2J, left) in Xiangya Cohort, in contrast to the results for DDX58. Secondly, in the imVigor210 cohort, IFN-I also showed significant positive correlation with CD8^+^ T cells and effector T cells (Fig. 2F, bottom). Differentially expressed genes in this cohort were significantly enriched in IFN-α and IFN-β-related pathways (Fig. S5A). Notably, YTHDF1 expression was negatively correlated with immune cell recruitment (Fig. 2I, right) and the activation of immune-related pathways (Fig. 2J, right), contrasting with the findings for DDX58. Lastly, in the TCGA-BLCA dataset, DDX58 expression was again positively correlated with immune cell recruitment and infiltration (Fig. S4A–D). YTHDF1 expression was found to be elevated in tumor tissues compared to adjacent normal tissues (Fig. 2K), and high YTHDF1 expression was associated with poor survival outcomes in bladder cancer patients (Fig. 2L). Survival analysis in the TCGA-BLCA cohort further supported these observations (Fig. S5B, C). Additionally, ISGs transcription are negatively correlated with YTHDF1 expression, and knockdown of YTHDF1 induces a spontaneous IFN signature in 5637 and T24 cells (Fig. S5D–F).

### YTHDF1 inhibits RIG-I expression and type I interferon release in bladder cancer through an m^6^A-dependent mechanism

**Fig. 3:**
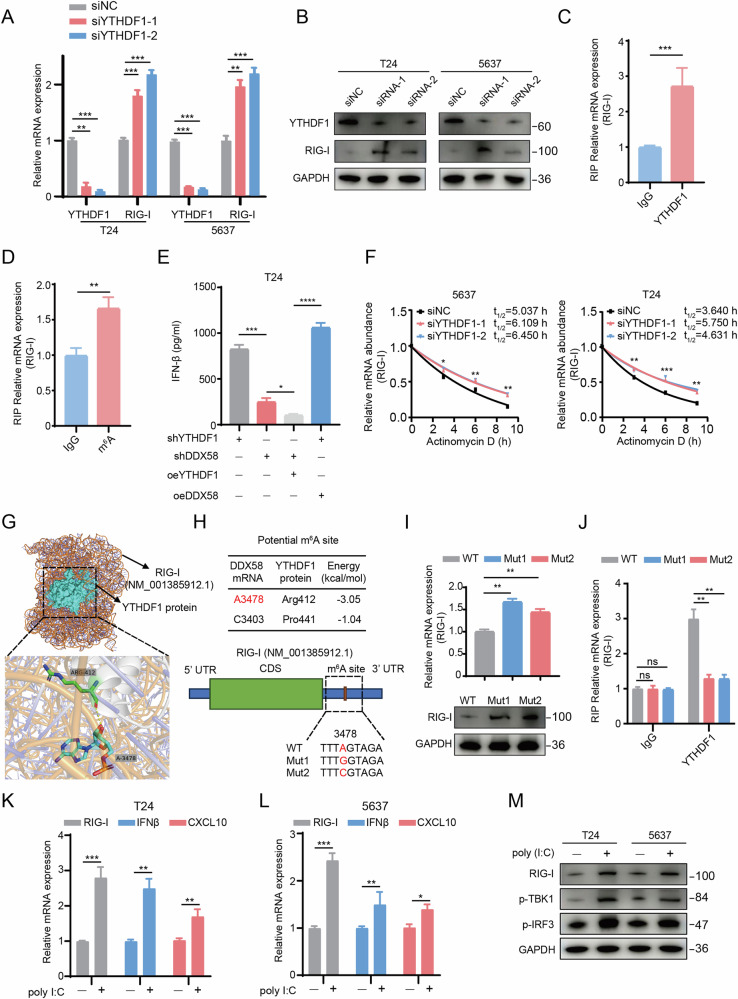
YTHDF1 inhibits RIG-I expression and type I interferon release in bladder cancer through an m^6^A-dependent mechanism. **A** RT-qPCR showing the differences in YTHDF1 and RIG-I mRNA expression after YTHDF1 knockdown in T24 and 5637 cells. **B** Western Blot detecting the differences in YTHDF1 and RIG-I protein expression after YTHDF1 knockdown in T24 and 5637 cells. **C** RIP-qPCR showing the differences in RIG-I mRNA binding to IgG and YTHDF1 antibodies. **D** RIP-qPCR showing the differences in RIG-I mRNA binding to IgG and m^6^A antibodies. **E** ELISA measuring IFN-β levels in the culture supernatant of T24 cells after knockdown or overexpression of specific molecules. **F** RNA degradation assays showed RIG-I RNA degradation following YTHDF1 knockdown in T24 and 5637 cells. AlphaFold 3 predictions of the interaction between YTHDF1 protein and RIG-I mRNA (**G**), along with possible m^6^A modification sites and point mutations at potential m^6^A modification sites on the YTHDF1 protein and RIG-I mRNA (**H**). **I** RT-qPCR and Western Blot detecting the differences in RIG-I mRNA and protein expression after point mutations. **J** RIP analysis demonstrating the binding relationship between YTHDF1 and mutant RIG-I mRNA. RT-qPCR detecting the differences in RIG-I, IFN-β, and CXCL10 mRNA expression in T24 (**K**) and 5637 (**L**) cells with or without poly (I:C) treatment. **M** Western Blot detecting the expression of type I interferon pathway-related proteins in T24 and 5637 cells with or without poly (I:C) treatment. *P < 0.05, **P < 0.01, ***P < 0.001, ****P < 0.0001.

Building upon the clinical phenotypic observations, we conducted cellular experiments and mechanistic investigations. We used two siRNAs to knock down YTHDF1 expression in T24 and 5637 bladder cancer cell lines and verified the results through RT-qPCR (Fig. 3A) and Western Blot (Fig. 3B). The findings showed that RIG-I expression was negatively correlated with YTHDF1 expression in both cell lines. Subsequently, we performed RNA immunoprecipitation (RIP) using IgG and YTHDF1 antibodies. RIP-qPCR revealed that the YTHDF1 protein binds to RIG-I mRNA (Fig. 3C). We then used IgG and m^6^A antibodies for RIP, and RIP-qPCR results demonstrated that the m^6^A antibody binds to RIG-I mRNA (Fig. 3D). These results suggest that YTHDF1 may regulate RIG-I mRNA expression in an m^6^A-dependent manner. We further examined the impact of overexpressing DDX58 or YTHDF1 on IFN-β release under YTHDF1 and DDX58 knockdown conditions using ELISA. The results indicated that the group with YTHDF1 knockdown and DDX58 overexpression had the highest IFN-β release, with statistically significant differences (Fig. 3E). This implies that YTHDF1 acts as an upstream regulator of DDX58. Additionally, we performed RNA degradation assays, which showed that following YTHDF1 knockdown in T24 and 5637 cells, RIG-I RNA degradation was significantly slowed (Fig. 3F). To identify the specific site where YTHDF1 interacts with RIG-I mRNA, we used Alphafold 3 for prediction (Fig. 3G). The results indicated that the A3478 site at the 3’ UTR end of RIG-I mRNA is the most likely m^6^A modification site. We then constructed two mutants targeting this site (Fig. 3H), and RNA and protein analysis confirmed that mutating A3478 led to increased RIG-I expression (Fig. 3I), validating this site as the m^6^A binding site for YTHDF1 on RIG-I mRNA. Additionally, RIP analysis revealed that YTHDF1 does not bind to the mutant RIG-I mRNAs (Fig. 3J). To further confirm the role of RIG-I activation in promoting immune responses, we treated T24 and 5637 cells with or without poly (I:C). RT-qPCR results showed that poly (I:C) treatment elevated the RNA levels of RIG-I, IFN-β, and CXCL10 in both cell lines, suggesting that RIG-I expression promotes the expression of genes related to immune infiltration (Fig. 3K, L). Western Blot analysis revealed similar results (Fig. 3M).

### Tumor-intrinsic YTHDF1 promotes MHC-I degradation and inhibits immune cell infiltration and differentiation

**Fig. 4:**
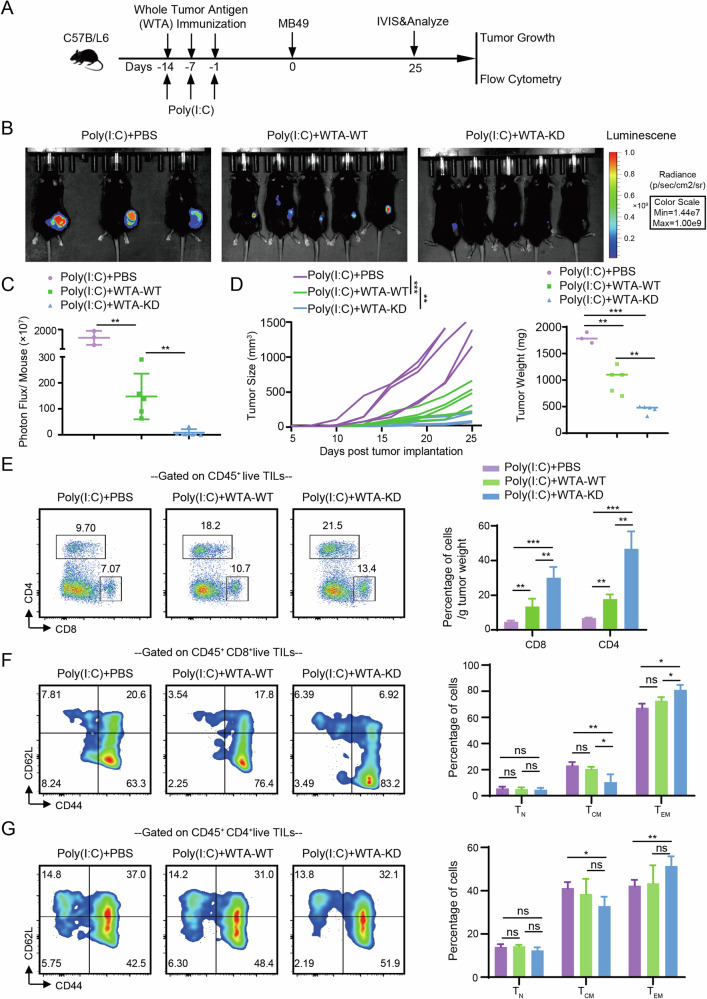
Tumor-intrinsic YTHDF1 promotes MHC-I degradation and inhibits immune cell infiltration and differentiation. **A** Experimental workflow diagram. **B** IVIS images showing tumor fluorescence intensity of mice in each treatment group at day 25. **C** Bar graph comparing tumor photon flux among different treatment groups. **D** Tumor growth curves and tumor weights of mice in each treatment group. **E** Representative flow cytometry plots and bar graphs depicting the proportions of CD4^+^ and CD8^+^ T cells infiltrating tumors per gram of tumor tissue in each treatment group. **F** Representative flow cytometry plots and bar graphs showing the differentiation status of CD8^+^ T cells infiltrating tumors in each treatment group. **G** Representative flow cytometry plots and bar graphs illustrating the differentiation status of CD4^+^ T cells infiltrating tumors in each treatment group. ns, P > 0.05, *P < 0.05, **P < 0.01, ***P < 0.001.

Next, we discovered that knocking down YTHDF1 significantly upregulated HLA-A expression in bladder cancer cells (Fig. S6A). This upregulation may be one of the reasons for the enhanced efficacy of immunotherapy. Furthermore, to determine whether the effect is lost in the absence of RIG-I, MAVS, or IFNAR, we assessed HLA-A expression in T24 cells with knockdowns of YTHDF1, RIG-I, MAVS, and IFNAR1. The results indicated that knocking down YTHDF1 led to increased HLA-A expression in T24 cells. Additionally, further knockdown of RIG-I, MAVS, or IFNAR1 in the context of YTHDF1 knockdown resulted in decreased HLA-A expression, although levels remained higher than in T24-shNC cells (Fig. S6B). To assess the immunogenicity of wild-type (WT) and Ythdf1-knockdown (KD) cells, mice were initially immunized with whole tumor antigens (WTA) from both groups. Fifteen days post-priming, they were challenged with MB49 cells. Briefly, WT or KD cells were sonicated to release WTA, and each lysate was combined with 100 μg of poly(I:C) and administered subcutaneously to mice three times at seven-day intervals. Following priming, C57BL/6 mice were challenged with MB49 cells for subsequent in vivo analysis (Fig. 4A). In vivo imaging results in mice showed that knocking down Ythdf1 led to a better immune response against MB49 bladder cancer (Fig. 4B-C). The tumor growth curves in mice also reflected the same outcome (Fig. 4D). Flow cytometry analysis of tumor-infiltrating lymphocytes revealed that Ythdf1 knockdown enhanced CD4^+^ T cell and CD8^+^ T cell infiltration per gram of tumor tissue in mice re-challenged with MB49 cells (Fig. 4E). Further analysis of CD8^+^ T cell (Fig. 4F) and CD4^+^ T cell (Fig. 4G) differentiation in treated mice indicated that the poly(I:C) + WTA-KD group exhibited a stronger tendency towards effector T cell (CD44^+^CD62L^-^) differentiation. The gating strategies can be seen in Fig. S6C. These findings indicate that YTHDF1 depletion suppressed the degradation of tumor antigens and MHC-I, ultimately enhancing tumor recognition and restoring tumor immune surveillance.

### Single-cell sequencing reveals distinct immune landscapes of tumor responses to ICI between the MRD and CD groups

**Fig. 5:**
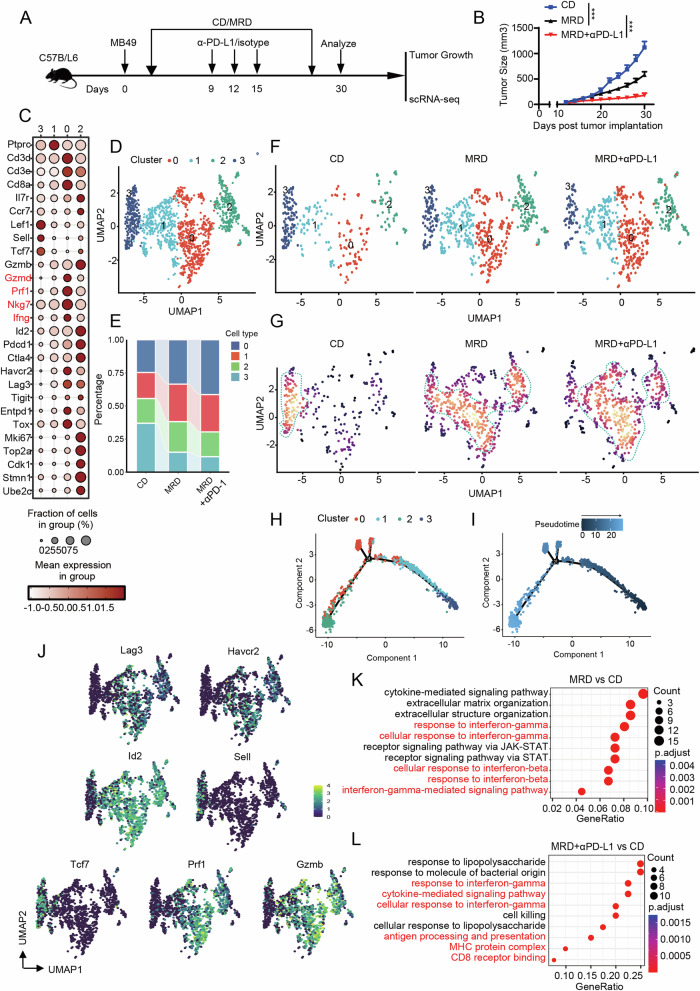
Methionine restriction promotes immune cell infiltration in bladder cancer and synergizes with immune checkpoint inhibitors. **A** Experimental workflow of single-cell sequencing. **B** Tumor growth curves of mice in each treatment group. **C** Differential expression of CD8^+^ T cell-associated molecules across different clusters. **D** UMAP plot of tumor-infiltrating T cell clusters. Proportions (**E**) and UMAP plot (**F**) of tumor-infiltrating T cell clusters across different treatment groups. **G** Density UMAP plot of tumor-infiltrating T cell clusters in different treatment groups. **H**, **I** Potential developmental trajectories of CD8^+^ T cell clusters in the tumor microenvironment across different clusters. **J** Expression levels of Lag3, Havcr2, Id2, Sell, Tcf7, Prf1, and GzmB in CD8^+^ T cell clusters indicated on the UMAP plot. **K** Enriched pathways comparing MRD group to CD group. **L** Enriched pathways comparing MRD + αPD-L1 group to CD group. ***P < 0.001. CD complete diet, MRD methionine restricted diet.

To better understand the impact of methionine regulation on YTHDF1 expression in tumor cells and its effect on tumor-infiltrating lymphocytes (TILs), we performed single-cell RNA sequencing (scRNA-seq) on tumor samples from CD, MRD, and MRD + αPD-L1 treated mice (Fig. 5A). The tumor growth curves of the mice in each treatment group are shown in Fig. 5B. Infiltration status of different cell types among various treated groups are shown in Fig. S7A. The results showed that there was no significant difference in the total amount of infiltrating macrophages and CD4^+^ T cells between the MRD and MRD + αPD-L1 groups compared to the CD group, however, there was a significant difference in the number of infiltrating CD8^+^ T cells (Fig. S7A–D). The marker genes for each cell subpopulation of CD8^+^ T cells are shown in Fig. 5C. Cell clustering analysis revealed four subpopulations of CD8^+^ T cells in the Uniform Manifold Approximation and Projection (UMAP) plot (Fig. 5D). Among these, the proportions of clusters 0 and 1 increased sequentially in the CD, MRD, and MRD + αPD-L1 groups, while the proportion of cluster 3 decreased sequentially. The proportion of cluster 2 was higher in the MRD and MRD + αPD-L1 groups compared to the CD group (Fig. 5E). The UMAP plots for each group are shown in Fig. 5F, and the density UMAP plot of cell distribution is shown in Fig. 5G.

To further elucidate the intrinsic developmental origins of CD8^+^ T cells, we applied the Monocle 2 algorithm to construct their potential developmental trajectories. We observed two major evolutionary branches, with clusters 0 and 2 positioned at the ends of different branches (Fig. 5H, I). This analysis demonstrated that CD8^+^ T cells infiltrating tumors on a methionine-restricted diet are more likely to differentiate into subpopulations with enhanced effector functions. Figure 5J shows the expression levels of Lag3, Havcr2, Id2, Sell, Tcf7, Prf1, and GzmB in CD8^+^ T cell clusters indicated on the UMAP plot. Subsequently, we conducted differential gene enrichment analysis on the sequencing results from each group. Enrichment analysis between the MRD group and CD group revealed significant enrichment in response to IFN-γ and response to IFN-β, suggesting that methionine restriction indeed affects the activation of the IFN-I pathway (Fig. 5K). Enrichment analysis between the MRD + αPD-L1 group and CD group (Fig. 5L) showed significant enrichment in cytokine-related pathways, antigen presentation, and MHC molecule binding, which corroborates our findings in Fig. 4.

### Deletion of YTHDF1 promotes immune cell infiltration in bladder cancer and synergizes with immune checkpoint inhibitors

**Fig. 6:**
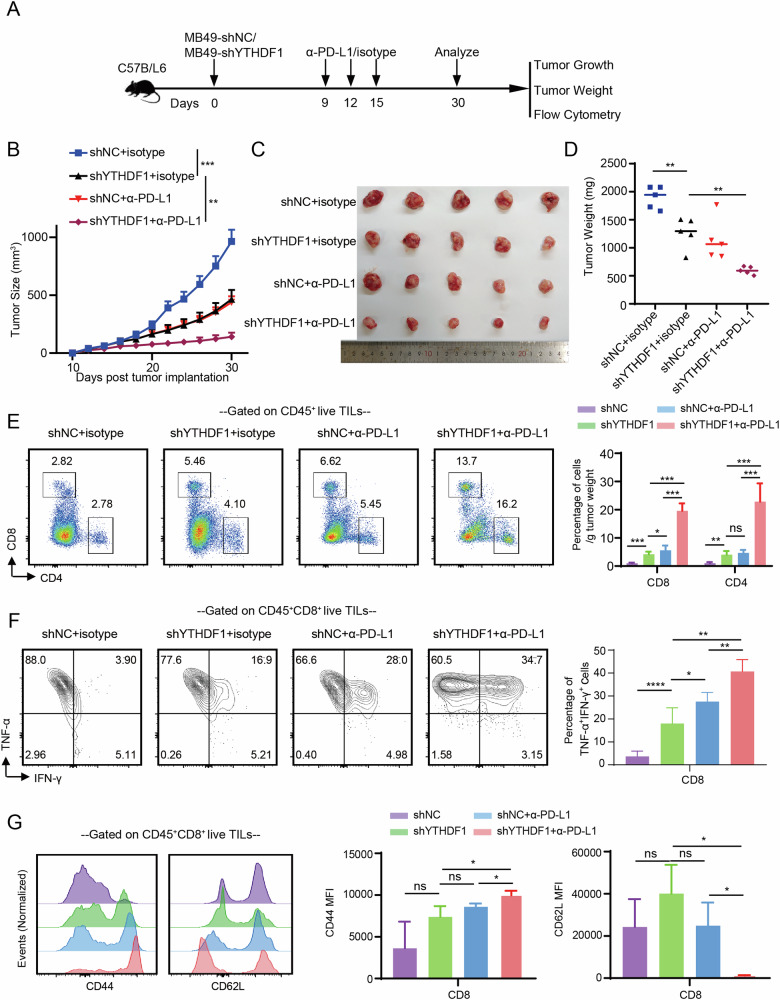
Deletion of YTHDF1 promotes immune cell infiltration in bladder cancer and synergizes with immune checkpoint inhibitors. **A** Diagram of the experimental workflow. Tumor growth curves (**B**), images of tumors (**C**), and tumor weights (**D**) for each treatment group. **E** Bar graphs and representative flow cytometry plots displaying the proportions of tumor-infiltrating CD4^+^ and CD8^+^ T cells per gram of tumor tissue in each treatment group. **F** Flow cytometry plots and bar graphs illustrating the proportions of CD8^+^ T cells producing IFN-γ and TNF-α in each treatment group. **G** Bar graphs and histograms showing the mean fluorescence intensity of CD44 and CD62L in CD8^+^ T cells across each treatment group. ns, P > 0.05, *P < 0.05, **P < 0.01, ***P < 0.001, ****P < 0.0001.

To further demonstrate that methionine impacts the tumor microenvironment (TME) through YTHDF1, we established a subcutaneous bladder cancer (BLCA) model in C57BL/6 mice using YTHDF1 knockdown and control mouse cell lines MB49 (Fig. 6A). Simultaneously, mice received either αPD-L1 or isotype control treatment on specific days during the experiment. We found that the deletion of YTHDF1 inhibited tumor growth in mice, and the combination of YTHDF1 KD with αPD-L1 treatment had a stronger anti-tumor effect (Fig. 6B). Upon reaching the ethical endpoint, we measured the tumor volume and weight and found that tumors in the YTHDF1 KD with αPD-L1 group were smaller and lighter (Fig. 6C, D). The tumors were digested into single-cell suspensions, and TILs were analyzed using flow cytometry. We observed that the YTHDF1 knockdown group had more CD8^+^ T cell infiltration per gram tumor tissue compared to the control group. This effect was even more pronounced in the YTHDF1 KD with αPD-L1 group, with the differences being statistically significant (Fig. 6E). Furthermore, the proportion of CD8^+^ TILs expressing IFN-γ and TNF-α was significantly higher in the YTHDF1 KD with αPD-L1 group compared to other groups (Fig. 6F). The intracellular staining FMO plots and gating strategies are shown in Fig. S6D. Additionally, YTHDF1 knockdown led to T cell differentiation, showing significantly higher expression of CD44 and lower expression of CD62L compared to the control group (Fig. 6G). Overall, our findings indicate that the deletion of YTHDF1 promotes the infiltration and differentiation of TILs, thereby enhancing the efficacy of αPD-L1 therapy.

### YTHDF1 interacts with eIF5B and promotes the translation of PD-L1

**Fig. 7:**
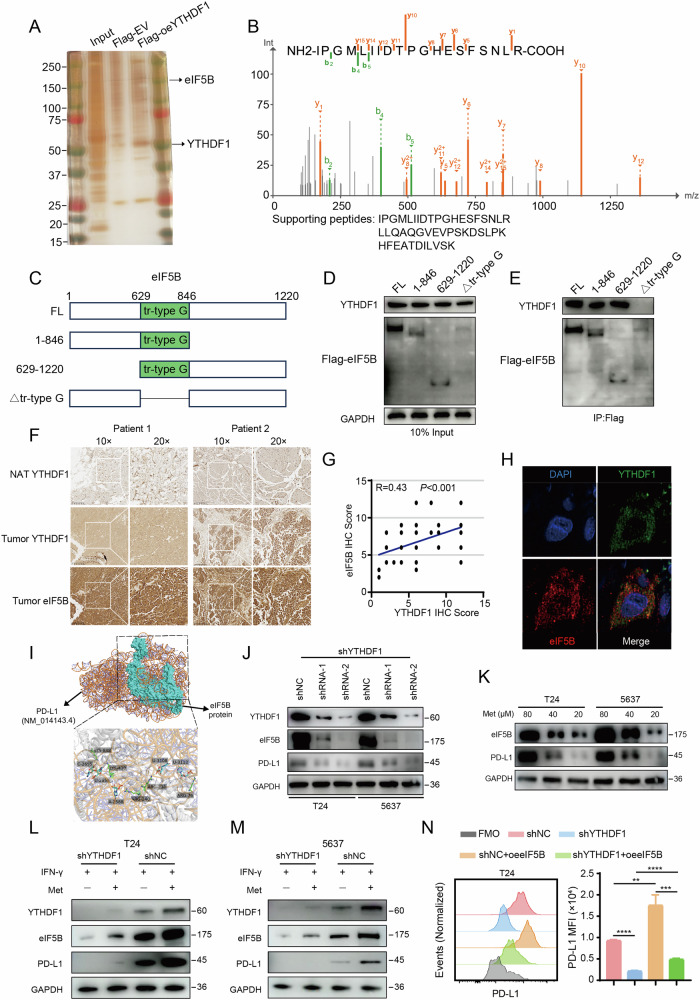
YTHDF1 interacts with eIF5B and promotes the translation of PD-L1. **A** Silver-stained gel revealed specific protein bands (highlighted with arrows) within the Flag-YTHDF1 immunoprecipitation sample, subsequently analyzed by LC–MS/MS. **B** Mass spectrometry identified eIF5B, which was precipitated from T24 cell lysates using a Flag antibody. **C** A schematic illustrates the full-length and truncated forms of eIF5B. **D**, **E** Co-immunoprecipitation assays identified the region of eIF5B interacting with YTHDF1. T24 cells transfected with various truncations underwent immunoprecipitation with an anti-Flag antibody targeting eIF5B, followed by immunoblotting using anti-Flag or anti-YTHDF1 antibodies. **F** Representative immunohistochemistry images of adjacent normal and tumor tissues from bladder cancer patients showing the correlation between YTHDF1 and eIF5B expression. **G** Correlation analysis of immunohistochemical scores for YTHDF1 and eIF5B in tumor tissues of bladder cancer patients.**H** Representative immunofluorescence images of tumor tissues from bladder cancer patients showing the correlation between endogenous YTHDF1 and eIF5B. **I** Alphafold 3 prediction of the binding between eIF5B protein and PD-L1 mRNA. **J** Western Blot analysis showing the expression of YTHDF1, eIF5B, and PD-L1 proteins after knocking down YTHDF1 in T24 and 5637 cells. **K** Western Blot analysis showing the expression of eIF5B and PD-L1 proteins after treating T24 and 5637 cells with different concentrations of methionine. (L-M) Western blot analysis of YTHDF1, eIF5B, and PD-L1 expression in T24 (**L**) and 5637 (**M**) cells with shYTHDF1 and shNC treatments, in the presence or absence of methionine and IFN-γ. **N** Representative histograms and bar graphs showing changes in PD-L1 expression in T24 cells after knocking down YTHDF1 and/or overexpressing eIF5B. **P < 0.01, ***P < 0.001, ****P < 0.0001. LC–MS/MS liquid chromatography-tandem mass spectrometry.

To investigate the molecular mechanisms by which YTHDF1 regulates the immune microenvironment, we stably overexpressed Flag-tagged YTHDF1 in T24 cells and performed immunoprecipitation, followed by liquid chromatography-tandem mass spectrometry (LC–MS/MS) to detect the global protein interactome of YTHDF1 (Fig. 7A). To identify proteins that specifically bind to YTHDF1, we compared the proteins bound to Flag-YTHDF1 with those bound to IgG. After excluding proteins that bound to IgG, the remaining proteins were ranked by MS score. Among these proteins, eIF5B was one of the candidate proteins that specifically associated with YTHDF1 (Fig. 7B). To confirm the interaction between YTHDF1 and eIF5B, we constructed truncated plasmids of eIF5B (Fig. 7C) and performed co-immunoprecipitation (co-IP) assays. The results showed that YTHDF1 primarily binds to the tr-type G domain of the eIF5B protein (Fig. 7D, E). Subsequently, we performed IHC staining on tumor tissues and adjacent normal tissues from bladder cancer patients, and found that the expression of YTHDF1 positively correlated with that of eIF5B (Fig. 7F, G). We further confirmed the interaction between endogenous YTHDF1 and eIF5B in bladder cancer patient tissues and T24 cells using immunofluorescence (Figs. 7H and  S8A).

Previous studies have shown that eIF5B can bind to the initiation site of PD-L1 mRNA and promote its translation [41]. Our research also found that YTHDF1 knockdown combined with αPD-L1 inhibitors sensitizes immunotherapy. Therefore, we used Alphafold 3 to predict the binding of eIF5B protein to PD-L1 mRNA, identifying five potential binding sites (Fig. 7I). Using Western Blot, we knocked down YTHDF1 expression in T24 and 5637 bladder cancer cell lines with two different shRNAs and detected the protein levels. The results showed that knockdown of YTHDF1 in both cell lines led to decreased expression of eIF5B and PD-L1 (Fig. 7J). We then examined the expression of eIF5B and PD-L1 proteins in human T24 cell lines in the presence or absence of IFN-γ and methionine in the culture medium. The results showed that IFN-γ promoted the expression of eIF5B and PD-L1 in both cell lines, but their expression decreased upon methionine deprivation and similar results were obtained when methionine deprivation was replaced by YTHDF1 knockdown (Fig. S8B). Next, we treated T24 and 5637 bladder cancer cell lines with different concentrations of methionine and found that the expression of eIF5B and PD-L1 proteins decreased with decreasing methionine concentration (Fig. 7K). Moreover, we treated YTHDF1 knockdown cells with methionine and IFN-γ. The results revealed that the expression of eIF5B and PD-L1 is dependent on the methionine-YTHDF1 axis. Specifically, after YTHDF1 knockdown, the addition of methionine led to a simultaneous increase in the expression of YTHDF1, eIF5B, and PD-L1. This phenomenon was confirmed in both T24 and 5637 cell lines (Fig. 7L, M). Conversely, overexpression of YTHDF1 in these cell lines increased eIF5B and PD-L1 expression (Fig. S8C). Additionally, overexpression of eIF5B rescued PD-L1 expression after YTHDF1 knockdown (Fig. S8D). Flow cytometry analysis of PD-L1 expression in T24 cells, where eIF5B was overexpressed after YTHDF1 knockdown, further validated these findings (Fig. 7N). Additionally, we knocked down IFNAR1 in T24 and 5637 cells, then used Western blotting to analyze the protein expression levels of YTHDF1, eIF5B, and PD-L1 under various culture conditions. The results showed that YTHDF1 and eIF5B expression were regulated exclusively by methionine, while PD-L1 expression increased in the presence of methionine. However, IFNAR1 knockdown led to a reduction in PD-L1 expression (Fig. S8E). In conclusion, these experiments demonstrate that methionine also regulates the tumor immune microenvironment through YTHDF1-eIF5B axis.

### Localized methionine deprivation in the bladder promotes immune cell infiltration and inhibits PD-L1 expression

The above results indicate that methionine deprivation can enhance the efficacy of immunotherapy against bladder cancer. However, methionine is an essential amino acid commonly found in protein-rich foods such as meat and eggs, and nutrition is crucial for patients with advanced cancer. Given the characteristics of bladder cancer, we considered whether local methionine deprivation could sensitize bladder cancer to immunotherapy. VNP20009, a genetically modified strain of Salmonella typhimurium lacking the msbB and purI genes, has been shown to be safe in several preclinical and Phase I clinical trials [42, 43]. However, due to its lack of significant anti-tumor effects at safe doses, many clinical trials were halted at Phase I. One of its mechanisms is to deprive tumors of methionine, thereby inhibiting their growth.

**Fig. 8:**
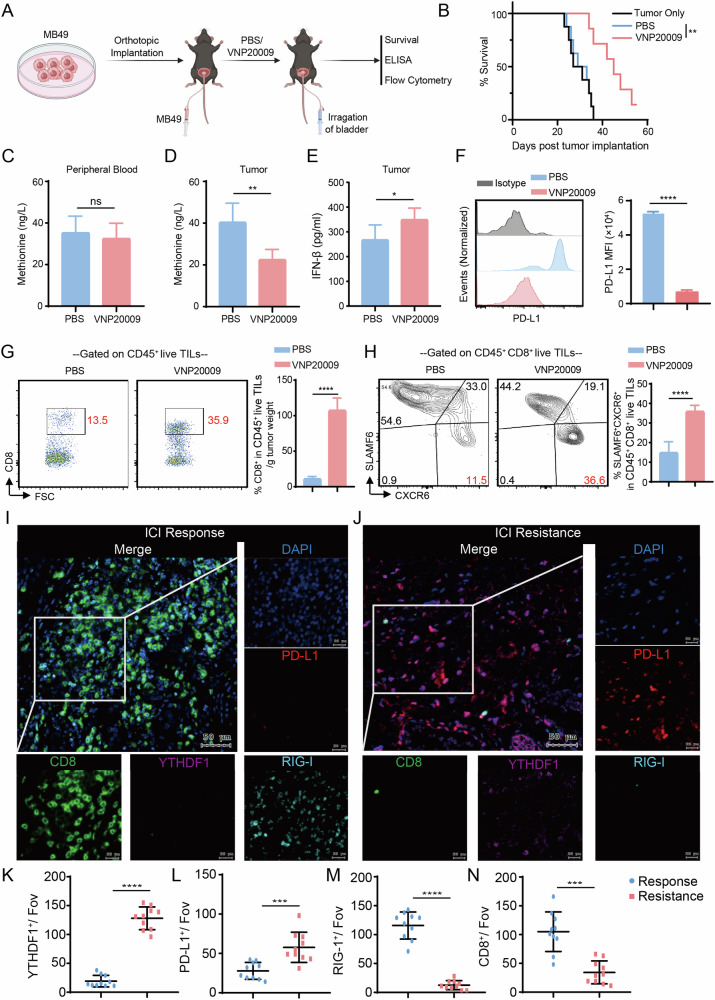
Localized methionine deprivation in the bladder promotes immune cell infiltration and inhibits PD-L1 expression. **A** Schematic of the mouse experiment. **B** Tumor growth curves of mice in different treatment groups. **C** ELISA detection of methionine levels in the peripheral blood of mice from different treatment groups. ELISA detection of methionine (**D**) and IFN-β (**E**) levels in the tumors of mice from different treatment groups. **F** Representative histograms and bar graphs showing the differences in PD-L1 expression in tumor cells across different treatment groups. **G** Representative flow cytometry plots and bar graphs showing the proportions of tumor-infiltrating CD8^+^ T cells per gram of tumor tissue in each treatment group. **H** Representative flow cytometry plots and bar graphs showing the proportions of SLAMF6^−^ and CXCR6^+^ CD8^+^ T cells in the tumors of each treatment group. Immunofluorescence images of CD8, YTHDF1, RIG-I, PD-L1, and DAPI staining in bladder cancer patients who responded (**I**) or were resistant (**J**) to immunotherapy from our center. **K**–**N** Bar graphs showing the statistical differences in immunofluorescence staining results between patients with different responses to immunotherapy. ns, P > 0.05, *P < 0.05, **P < 0.01, ***P < 0.001, ****P < 0.0001.

As shown in Fig. 8A, we performed bladder instillation of VNP20009 or PBS in mice with orthotopic MB49 bladder tumors and observed tumor growth. The results showed that bladder instillation of VNP20009 significantly prolonged the survival of tumor-bearing mice (Fig. 8B). Additionally, ELISA results indicated that peripheral blood methionine levels in the VNP20009-treated group were not significantly different from those in the PBS-treated group (Fig. 8C). However, methionine levels in the tumor lysate supernatant were significantly reduced in the VNP20009-treated group compared to the PBS-treated group (Fig. 8D), while IFN-β levels were significantly increased (Fig. 8E), accompanied by a decrease in PD-L1 expression on the surface of tumor cells (Fig. 8F). These results are consistent with our previous findings. Analysis of tumor-infiltrating lymphocytes revealed that more CD8^+^ T cells infiltrated per gram tumor tissue of mice treated with VNP20009 compared to the PBS-treated group (Fig. 8G), and these cells were more likely to differentiate into the effector SLAMF6^−^CXCR6^+^ subset (Fig. 8H). These experimental results demonstrate that bladder instillation of VNP20009 is an effective local methionine deprivation method that enhances bladder cancer immunotherapy.

Clinically, we performed multicolor immunofluorescence staining on tumor tissues from bladder cancer patients who responded to or resisted immunotherapy. The results showed that in the responsive group, tumors exhibited high expression of RIG-I, abundant infiltration of CD8^+^ T cells, and low expression of YTHDF1 and PD-L1 (Fig. 8I). In contrast, tumors from the resistant group showed low expression of RIG-I, fewer infiltrating CD8^+^ T cells, and high expression of YTHDF1 and PD-L1 (Fig. 8J), with statistically significant differences (Fig. 8K–N). Figure S9 summarizes the schematic diagram of this study.

## Discussions

Unlike normal cells, cancer cells possess the ability for unlimited proliferation and can disrupt normal cellular tissues [38, 44–47]. To meet the demands of proliferation, cancer cells enhance metabolic efficiency and acquire more nutrients, with amino acids serving as a crucial energy source, providing a cornerstone for cancer cell development and offering an entry point for preventing cancer progression [25, 29, 48, 49]. The concept of amino acid imbalance (AAI) emerged in the 1950s, initiating exploration into altering amino acid intake to influence cancer progression [50–52]. Methionine is an essential amino acid and the most variable metabolite in human plasma [30]. Consequently, methionine becomes a primary candidate target for amino acid depletion therapy, especially in tumors driven by epigenetic modifications [26, 27]. Experiments have shown that restricting methionine intake can inhibit the growth of tumors such as triple-negative breast cancer, colorectal cancer, sarcomas, and gliomas, and suppress cancer cell metastasis [26, 27, 30, 53].

Certainly, therapies that target amino acid metabolism often inadvertently suppress the function of immune cells within the tumor microenvironment, as these cells utilize many of the same metabolic pathways as tumor cells [54]. Our experiments robustly demonstrated that methionine restriction diet (MRD) enhances antitumor efficiency by activating IFN-I signaling and m^6^A-dependent mechanisms, thereby improving the clinical outcomes of immune checkpoint blockade (ICB) therapy. Notably, malnutrition symptoms frequently occur in cancer patients, particularly those with advanced tumors. Methionine, found abundantly in high-protein foods like meat, milk, and beans, plays a crucial role. Limiting these diets may negatively impact tumor treatment and how to balance its advantages and disadvantages is a question worthy of thinking. Therefore, based on the unique treatment method for bladder cancer (bladder instillation), we utilized VNP20009, an oncolytic virus, in murine bladder instillation experiments. The crucial mechanism involved methionine deprivation, as evidenced by the reduction of methionine levels in tumors without significant changes in peripheral blood, thereby avoiding malnutrition in advanced bladder cancer patients due to inadequate amino acid intake.

In this study, we demonstrated that bladder cancer cells suppress IFN-I signaling via the RIG-I-MAVS pathway by ingesting methionine, which also acts as a methylating medium, upregulating the cytoplasmic m^6^A reader YTHDF1. Knockdown of YTHDF1 significantly enhanced bladder cancer cells’ response to immune checkpoint inhibitors. RNA-seq further confirmed that YTHDF1 loss upregulates the RIG-I-mediated IFN-I signal. Using WTA as an immune vaccine, we investigated how YTHDF1 knockdown affects the immunogenicity of MB49 cells, including MHC-I expression. Mass spectrometry showed that YTHDF1 enhances its expression by binding to eIF5B. Our findings suggest that the YTHDF1-RIG-I axis is crucial in bladder cancer pathogenesis and mediates the immune response to local methionine deprivation (VNP20009). This strategy, similar to BCG bladder instillation, targets the tumor microenvironment with minimal systemic effects. Combining VNP20009 with BCG may enhance immunotherapy by amplifying immune activation and cell infiltration, offering a promising research direction. Multiplex immunofluorescence staining of bladder cancer immunotherapy patients’ tissues also supported this conclusion.

However, in this study, we encountered some unresolved issues. We found that methionine promotes the expression of YTHDF1, leading to the downregulation of DDX58 expression. Previously, it was thought that in the cytoplasm, the m^6^A-binding protein YTHDF1 promotes the translation of m^6^A-modified mRNA, while YTHDF2 facilitates the degradation of m^6^A-modified mRNA. This finding seems to contradict the previous understanding. Nevertheless, there are also studies indicating that YTHDF1 may inhibit mRNA translation under certain conditions. Li et al. [55] reported that YTHDF1 promotes mRNA degradation through liquid-liquid phase separation and interact with AGO2. Zaccara et al. [56] reported that the degradation of m^6^A-modified mRNA is driven by the combined activity of YTHDF1/2/3 proteins. Therefore, we considered the following two possibilities: Firstly, methionine endows intracellular free RNA with methylation, leading to m^6^A modification. The modified RNA binds with YTHDF1 to form a complex, which, due to its spatial conformation, cannot be recognized by RIG-I. Consequently, the complex loses its ability to induce RIG-I expression, resulting in reduced RIG-I levels and the inactivation of the corresponding pathway. Secondly, research has shown that m^6^A modification at the 5′ end of RNA promotes its reverse transcription, while m^6^A modification at the 3′ end reduces RNA stability. In our study, we predicted and confirmed that the m^6^A site of DDX58 mRNA bound by YTHDF1 is located at its 3′ end, which could explain why YTHDF1 leads to the downregulation of DDX58 expression. However, we did not conduct detailed experiments to confirm this, which is a limitation of our study. Additionally, we lack a clinical cohort from our center with sufficient follow-up time to evaluate the prognosis of bladder cancer immunotherapy, preventing us from validating the effect of methionine on immunotherapy outcomes from a clinical perspective.

In conclusion, our research shows that in bladder cancer immunotherapy, methionine promotes YTHDF1 upregulation through mRNA methylation. This upregulation allows YTHDF1 to interact with DDX58, leading to its degradation and a subsequent reduction in IFN-I expression. Additionally, YTHDF1 binds to eIF5B, enhancing PD-L1 mRNA translation. These mechanisms contribute to immunotherapy resistance. Importantly, localized methionine deprivation within the bladder boosts immune cell infiltration and effector functions, potentially overcoming this resistance.

## Supplementary information

SUPPLEMENTAL MATERIALS

Table S1

Table S2

Table S3

Original WB

## Data Availability

The datasets generated or analyzed during the current study are available in the TCGA-BLCA repository with the link: https://portal.gdc.cancer.gov/, the pan-cancer GEO database with the link: https://www.ncbi.nlm.nih.gov/geo/, the IMvigor210 cohort with the link: http://researchpub.Gene.com/imvigor210corebiologies/. The Xiangya cohort, bulk RNA and single-cell RNA sequencing data generated or analyzed during the current study are available from the corresponding author on reasonable request.

## References

[1] Yu A, Hu J, Fu L, Huang G, Deng D, Zhang M, et al. Bladder cancer intrinsic LRFN2 drives anticancer immunotherapy resistance by attenuating CD8(+) T cell infiltration and functional transition. J Immunother Cancer. 2023;11:e007230. 37802603 10.1136/jitc-2023-007230 PMC10565151

[2] Lopez-Beltran A, Cookson MS, Guercio BJ, Cheng L. Advances in diagnosis and treatment of bladder cancer. BMJ 2024;384:e076743. 38346808 10.1136/bmj-2023-076743

[3] Siefker-Radtke AO, Desai M. Evolution of front-line immunotherapy for metastatic urothelial cancer. Lancet Oncol. 2024;25:2–3. 38101432 10.1016/S1470-2045(23)00633-2

[4] Koti M, Robert Siemens D. A step closer to predicting progression after bacillus calmette-guérin immunotherapy in high-risk non-muscle-invasive bladder cancer. Eur Urol. 2023;84:447–8. 37400353 10.1016/j.eururo.2023.06.017

[5] Griffin J. TIME for a change? Multimarker assessment of the tumour immune microenvironment and metastatic site biopsy to improve immunotherapy response prediction in muscle-invasive bladder cancer. Eur Urol. 2023;83:143–4. 36443154 10.1016/j.eururo.2022.11.008

[6] Necchi A, Faltas BM, Slovin SF, Meeks JJ, Pal SK, Schwartz LH, et al. Immunotherapy in the treatment of localized genitourinary cancers. JAMA Oncol. 2023;9:1447–54. 37561425 10.1001/jamaoncol.2023.2174 PMC11429659

[7] Hwang TJ, Davies BJ, Preston MA. Advancing patient-centered outcomes and equity in clinical trials for BCG-unresponsive nonmuscle invasive bladder cancer. JAMA Oncol. 2023;9:1491–2. 37676669 10.1001/jamaoncol.2023.3304

[8] Hayne D, Redfern A. The evolving role of locally delivered checkpoint inhibitors in non-muscle-invasive bladder cancer. Eur Urol. 2022;82:611–2. 36153248 10.1016/j.eururo.2022.08.032

[9] Ranti D, Bieber C, Wang YS, Sfakianos JP, Horowitz A. Natural killer cells: unlocking new treatments for bladder cancer. Trends Cancer. 2022;8:698–710. 35581130 10.1016/j.trecan.2022.03.007

[10] Su W, Qiu W, Li SJ, Wang S, Xie J, Yang QC, et al. A dual-responsive STAT3 inhibitor nanoprodrug combined with oncolytic virus elicits synergistic antitumor immune responses by igniting pyroptosis. Adv Mater. 2023;35:e2209379. 36545949 10.1002/adma.202209379

[11] Moreo E, Uranga S, Picó A, Gómez AB, Nardelli-Haefliger D, Del Fresno C, et al. Novel intravesical bacterial immunotherapy induces rejection of BCG-unresponsive established bladder tumors. J Immunother Cancer. 2022;10:e004325. 10.1136/jitc-2021-004325 PMC9252205 35781395

[12] Fu J, Yu A, Xiao X, Tang J, Zu X, Chen W, et al. CD4(+) T cell exhaustion leads to adoptive transfer therapy failure which can be prevented by immune checkpoint blockade. Am J Cancer Res. 2020;10:4234–50. 33414997 PMC7783768

[13] Yu A, Xu X, Pang Y, Li M, Luo J, Wang J, et al. PD-L1 expression is linked to tumor-infiltrating T-cell exhaustion and adverse pathological behavior in pheochromocytoma/paraganglioma. Lab Investig. 2023;103:100210. 37406931 10.1016/j.labinv.2023.100210

[14] Yu A, Fu J, Yin Z, Yan H, Xiao X, Zou D, et al. Continuous expression of interferon regulatory factor 4 sustains CD8(+) T cell Immunity against Tumor. Research. 2023;6:0271. 38178902 10.34133/research.0271 PMC10765897

[15] Taffoni C, Marines J, Chamma H, Guha S, Saccas M, Bouzid A, et al. DNA damage repair kinase DNA-PK and cGAS synergize to induce cancer-related inflammation in glioblastoma. EMBO J. 2023;42:e111961. 36574362 10.15252/embj.2022111961 PMC10068334

[16] Jiang Y, Zhang H, Wang J, Chen J, Guo Z, Liu Y, et al. Exploiting RIG-I-like receptor pathway for cancer immunotherapy. J Hematol Oncol. 2023;16:8. 36755342 10.1186/s13045-023-01405-9 PMC9906624

[17] Bordon Y. Disturbance of cytoskeleton primes RIG-I-like receptors. Nat Rev Immunol. 2022;22:654–5. 36175502 10.1038/s41577-022-00789-y PMC9521866

[18] Li Z, Zhou Y, Jia K, Yang Y, Zhang L, Wang S, et al. JMJD4-demethylated RIG-I prevents hepatic steatosis and carcinogenesis. J Hematol Oncol. 2022;15:161. 36333807 10.1186/s13045-022-01381-6 PMC9636772

[19] Lou Q, Jiang K, Xu Q, Yuan L, Xie S, Pan Y, et al. The RIG-I-NRF2 axis regulates the mesenchymal stromal niche for bone marrow transplantation. Blood. 2022;139:3204–21. 35259210 10.1182/blood.2021013048

[20] Murayama T, Nakayama J, Jiang X, Miyata K, Morris AD, Cai KQ, et al. Targeting DHX9 triggers tumor-intrinsic interferon response and replication stress in small cell lung cancer. Cancer Discov. 2024;14:468–91. 38189443 10.1158/2159-8290.CD-23-0486 PMC10905673

[21] Chiappinelli KB. Targeting the DHX9 RNA helicase to induce antitumor immunity in small-cell lung cancer. Cancer Discov. 2024;14:389–91. 38426559 10.1158/2159-8290.CD-23-1523

[22] Guil S, Esteller M. PRC2 loss and dnmt inhibition boost viral mimicry in cancer. Cancer Discov. 2022;12:2020–2. 36052503 10.1158/2159-8290.CD-22-0733

[23] Elmaleh-Sachs A, Schwartz JL, Bramante CT, Nicklas JM, Gudzune KA, Jay M. Obesity management in adults: a review. JAMA 2023;330:2000–15. 38015216 10.1001/jama.2023.19897 PMC11325826

[24] Kang JS. Dietary restriction of amino acids for cancer therapy. Nutr Metab. 2020;17:20. 10.1186/s12986-020-00439-x PMC7071719 32190097

[25] Li F, Liu P, Mi W, Li L, Anderson NM, Lesner NP, et al. Blocking methionine catabolism induces senescence and confers vulnerability to GSK3 inhibition in liver cancer. Nat Cancer. 2024;5:131–46. 38168934 10.1038/s43018-023-00671-3 PMC11277537

[26] Lu W, Luo Y. Methionine restriction sensitizes cancer cells to immunotherapy. Cancer Commun. 2023;43:1267–70. 10.1002/cac2.12492 PMC10631477 37803877

[27] Ji M, Xu X, Xu Q, Hsiao YC, Martin C, Ukraintseva S, et al. Methionine restriction-induced sulfur deficiency impairs antitumour immunity partially through gut microbiota. Nat Metab. 2023;5:1526–43. 37537369 10.1038/s42255-023-00854-3 PMC10513933

[28] Guo R, Liang JH, Zhang Y, Lutchenkov M, Li Z, Wang Y, et al. Methionine metabolism controls the B cell EBV epigenome and viral latency. Cell Metab. 2022;34:1280–97.e9. 36070681 10.1016/j.cmet.2022.08.008 PMC9482757

[29] Bian Y, Li W, Kremer DM, Sajjakulnukit P, Li S, Crespo J, et al. Cancer SLC43A2 alters T cell methionine metabolism and histone methylation. Nature. 2020;585:277–82. 32879489 10.1038/s41586-020-2682-1 PMC7486248

[30] Wei F, Locasale JW. Methionine restriction and antitumor immunity. Trends Cancer. 2023;9:705–6. 37517954 10.1016/j.trecan.2023.07.008 PMC10458792

[31] Morehead LC, Garg S, Wallis KF, Simoes CC, Siegel ER, Tackett AJ, et al. Increased response to immune checkpoint inhibitors with dietary methionine restriction in a colorectal cancer model. Cancers. 2023;15:4467. 10.3390/cancers15184467 PMC10526448 37760436

[32] Ni Z, Sun P, Zheng J, Wu M, Yang C, Cheng M, et al. JNK signaling promotes bladder cancer immune escape by regulating METTL3-mediated m6A modification of PD-L1 mRNA. Cancer Res. 2022;82:1789–802. 35502544 10.1158/0008-5472.CAN-21-1323

[33] Guimarães-Teixeira C, Lobo J, Miranda-Gonçalves V, Barros-Silva D, Martins-Lima C, Monteiro-Reis S, et al. Downregulation of m(6) A writer complex member METTL14 in bladder urothelial carcinoma suppresses tumor aggressiveness. Mol Oncol. 2022;16:1841–56. 35048498 10.1002/1878-0261.13181 PMC9067151

[34] Xu P, Ge R. Roles and drug development of METTL3 (methyltransferase-like 3) in anti-tumor therapy. Eur J Med Chem. 2022;230:114118. 35063732 10.1016/j.ejmech.2022.114118

[35] Lv L, Wei Q, Zhang J, Dong Y, Shan Z, Chang N, et al. IGF2BP3 prevent HMGB1 mRNA decay in bladder cancer and development. Cell Mol Biol Lett. 2024;29:39. 38504159 10.1186/s11658-024-00545-1 PMC10949762

[36] Liu P, Fan B, Othmane B, Hu J, Li H, Cui Y, et al. m(6)A-induced lncDBET promotes the malignant progression of bladder cancer through FABP5-mediated lipid metabolism. Theranostics. 2022;12:6291–307. 36168624 10.7150/thno.71456 PMC9475447

[37] Lin W, Chen L, Zhang H, Qiu X, Huang Q, Wan F, et al. Tumor-intrinsic YTHDF1 drives immune evasion and resistance to immune checkpoint inhibitors via promoting MHC-I degradation. Nat Commun. 2023;14:265. 36650153 10.1038/s41467-022-35710-7 PMC9845301

[38] Li L, He S, Liao B, Wang M, Lin H, Hu B, et al. Orally administrated hydrogel harnessing intratumoral microbiome and microbiota-related immune responses for potentiated colorectal cancer treatment. Research. 2024;7:0364. 38721274 10.34133/research.0364 PMC11077293

[39] Wu J, Liu N, Chen J, Tao Q, Li Q, Li J, et al. The tricarboxylic acid cycle metabolites for cancer: friend or enemy. Research (Wash D C). 2024;7:0351. 38867720 10.34133/research.0351 PMC11168306

[40] Tsukamoto Y, Hiono T, Yamada S, Matsuno K, Faist A, Claff T, et al. Inhibition of cellular RNA methyltransferase abrogates influenza virus capping and replication. Science. 2023;379:586–91. 36758070 10.1126/science.add0875

[41] Suresh S, Chen B, Zhu J, Golden RJ, Lu C, Evers BM, et al. eIF5B drives integrated stress response-dependent translation of PD-L1 in lung cancer. Nat Cancer. 2020;1:533–45. 32984844 10.1038/s43018-020-0056-0 PMC7511089

[42] Yang H, Yang S, Guo Q, Sheng J, Mao Z. ATP-responsive manganese-based bacterial materials synergistically activate the cGAS-STING pathway for tumor immunotherapy. Adv Mater. 2024;36:e2310189. 38414097 10.1002/adma.202310189

[43] Wu L, Du Z, Li L, Qiao L, Zhang S, Yin X, et al. Camouflaging attenuated Salmonella by cryo-shocked macrophages for tumor-targeted therapy. Signal Transduct Target Ther. 2024;9:14. 38195682 10.1038/s41392-023-01703-1 PMC10776584

[44] Lee YC, Lam HM, Rosser C, Theodorescu D, Parks WC, Chan KS. The dynamic roles of the bladder tumour microenvironment. Nat Rev Urol. 2022;19:515–33. 35764795 10.1038/s41585-022-00608-y PMC10112172

[45] Meng H, Yu Y, Xie E, Wu Q, Yin X, Zhao B, et al. Hepatic HDAC3 regulates systemic iron homeostasis and ferroptosis via the Hippo signaling pathway. Research. 2023;6:0281. 38034086 10.34133/research.0281 PMC10687581

[46] Xiao C, Li J, Hua A, Wang X, Li S, Li Z, et al. Hyperbaric oxygen boosts antitumor efficacy of copper-diethyldithiocarbamate nanoparticles against pancreatic ductal adenocarcinoma by regulating cancer stem cell metabolism. Research. 2024;7:0335. 38766644 10.34133/research.0335 PMC11100349

[47] Zhou J, Wei Z, Yang C, Jia D, Pan B, Zeng Y, et al. APE1 promotes radiation resistance against radiation-induced pyroptosis by inhibiting the STING pathway in lung adenocarcinoma. Transl Oncol. 2023;36:101749. 37544034 10.1016/j.tranon.2023.101749 PMC10424251

[48] Vellai T. How the amino acid leucine activates the key cell-growth regulator mTOR. Nature. 2021;596:192–4. 34290413 10.1038/d41586-021-01943-7

[49] Fang W, Jiang L, Zhu Y, Yang S, Qiu H, Cheng J, et al. Methionine restriction constrains lipoylation and activates mitochondria for nitrogenic synthesis of amino acids. Nat Commun. 2023;14:2504. 37130856 10.1038/s41467-023-38289-9 PMC10154411

[50] Deng M, Yu J, Blackmond DG. Symmetry breaking and chiral amplification in prebiotic ligation reactions. Nature. 2024;626:1019–24. 38418914 10.1038/s41586-024-07059-y

[51] Grandison RC, Piper MD, Partridge L. Amino-acid imbalance explains extension of lifespan by dietary restriction in Drosophila. Nature. 2009;462:1061–4. 19956092 10.1038/nature08619 PMC2798000

[52] Rechcigl M Jr., Berger S, Loosli JK, Williams HH. Effect of “amino-acid imbalance” on growth and vitamin A storage in the white rat. Nature. 1959;184:1404. 10.1038/1841404a0 14436765

[53] Dai Z, Mentch SJ, Gao X, Nichenametla SN, Locasale JW. Methionine metabolism influences genomic architecture and gene expression through H3K4me3 peak width. Nat Commun. 2018;9:1955. 29769529 10.1038/s41467-018-04426-y PMC5955993

[54] Li J, Zheng C, Mai Q, Huang X, Pan W, Lu J, et al. Tyrosine catabolism enhances genotoxic chemotherapy by suppressing translesion DNA synthesis in epithelial ovarian cancer. Cell Metab. 2023;35:2044–59.e8. 37890478 10.1016/j.cmet.2023.10.002

[55] Li J, Chen K, Dong X, Xu Y, Sun Q, Wang H, et al. YTHDF1 promotes mRNA degradation via YTHDF1-AGO2 interaction and phase separation. Cell Prolif. 2022;55:e13157. 34821414 10.1111/cpr.13157 PMC8780909

[56] Zaccara S, Jaffrey SR. A unified model for the function of YTHDF proteins in regulating m(6)A-modified mRNA. Cell. 2020;181:1582–95.e18. 32492408 10.1016/j.cell.2020.05.012 PMC7508256

