# Supplementary material for: Methionine-driven YTHDF1 expression facilitates bladder cancer progression by attenuating RIG-I-modulated immune responses and enhancing the eIF5B-PD-L1 axis
Source: Cell Death Differ. 2024 Dec 13;32(4):776–91. doi: 10.1038/s41418-024-01434-y (PMC11982326; doi:10.1038/s41418-024-01434-y)
Supplement: Supplementary file 1 — SUPPLEMENTAL MATERIALS [file 41418_2024_1434_MOESM1_ESM.pdf]

Materials and methods

Cell culture and treatment

T24, 5637, UMUC-3, MB49 cell lines were obtained from American Type Culture Collection (ATCC, Manassas, VA, USA) and identified through STR profiling, with testing conducted to ensure mycoplasma-free status. The above cells were cultured in DMEM medium (Gibco) containing 10% FBS. The drugs used for cell treatment include Trifluoromethyl-tubercidin (TFMT) (HY-156048, MCE), methionine (M1952, Abmole), homocysteine (M45193, Abmole), S-adenosyl methionine (SAM) (HY-B0617A, MCE), and S-adenosylhomocysteine (SAH) (HY-19528, MCE).

Stable cell line construction

The shRNA and sgRNA plasmids were constructed by GK Biotechnology (Shanghai, China), the siRNAs were constructed by RIBO Biotechnology (Guangzhou, China). The detailed sequence can be found in Table S1. Lentiviral particles were generated using a packaging system and transfected into target cells according to standard protocols. Following transfection, cells were selected with puromycin to ensure stable integration of the lentivirus.

qRT-PCR, RNA-seq and clinical cohorts' analysis

Total RNA from cells was extracted using TRIzol reagent and subjected to qRT-PCR and RNA sequencing. The RNA-seq results can be find in Table S3. cDNA was synthesized using the PrimeScript RT Master Mix Kit (Takara, Tokyo, Japan), followed by real-time PCR performed with GoTaq qPCR Master Mix (Promega, Madison, USA) according to the manufacturer's guidelines. Gene expression data were normalized to GAPDH levels, and the primer sequences used are provided in Table S2. For the analysis of RNA-seq data and public databases, the GISTIC algorithm was utilized to process CNV data. Xiangya Cohort was described as we previously reported(1).

Amino Acid Addition and Restriction

The 20 essential and non-essential amino acids were purchased from Abmole (Cat:M49817) and

dissolved and added to the culture medium according to the instructions. Methionine-deficient DMEM medium was purchased from Beyotime (Cat:C0891), and BLCA cells were subjected to respective tests after treatment with methionine-restricted culture medium or control medium for 12 hours. For in vivo methionine restriction diet, diets containing 0.12% methionine were obtained from Dyets Company (Wuxi, China).

RNA Stability Assay

Under different culture or treatment conditions, cells were treated with 5 mg/mL actinomycin D (SBR00013, Sigma-Aldrich). Harvesting of the cells occurred at various time intervals. qRT-PCR was employed to assess the relative expression levels of mRNA at these time points.

Tissue specimens

The BLCA samples were obtained from First Affiliated Hospital of Sun Yat-sen University and Xiangya Hospital of Central South University. In addition to patients diagnosed with bladder cancer through pathology, all other patients were excluded. All experiments passed the Ethics Committee of First Affiliated Hospital of Sun Yat-sen University and Xiangya Hospital of Central South University.

ELISA

The ELISA kits used in the experiment are as follows: Human IFN-β ELISA Kit (cat: E-EL-H0085, Elabscience); Mouse IFN-β ELISA Kit (cat: E-EL-M0033, Elabscience); Met ELISA Kit (cat: MM-92614902, Meimian). The supernatant of tumor cell cultures or tissue lysates was collected and used for subsequent ELISA experiments. Poly (I:C) was purchased from Invivogen. All experiments were conducted according to the manufacturer's instructions.

Western blot

Briefly, RIPA buffer was used to lyse the samples, followed by elution with 2×SDS buffer, resolution on SDS-polyacrylamide gels, and subsequent transfer to polyvinylidene fluoride membranes. Primary antibodies were then applied to the membranes, which were subsequently incubated with horseradish peroxidase (HRP)-conjugated secondary antibodies. The primary antibodies used are as follows: RIG-I (20566-1-AP, Proteintech), MDA5 (21775-1-AP,

Proteintech), MAVS (66911-1-Ig, Proteintech), TBK1 (3504, Cell Signaling Technology), p-TBK1 (5483, Cell Signaling Technology), IRF3 (66670-1-Ig, Proteintech), p-IRF3 (29528-1-AP, Proteintech), GAPDH (60004-1-Ig, Proteintech), Histone H3 (9715, Cell Signaling Technology), H3K4me3 (9751, Cell Signaling Technology), H3K9me3 (13969, Cell Signaling Technology), H3K27me3 (9733, Cell Signaling Technology), YTHDF1 (17479-1-AP, Proteintech), Flag (YM3809, Immunoway), eIF5B (13527-1-AP, Proteintech), PD-L1 (28076-1-AP, Proteintech). Signal detection and visualization were achieved through enhanced chemiluminescence assay.

LDH assay

Activated human T cells from a healthy donor were co-cultured with T24 cells in a 12-well plate for 12 hours, either in normal medium or methionine-free medium. The supernatants were collected for the LDH assay (C0016, Beyotime) following the manufacturer's protocol.

m^{6}A dot blot

TRIzol reagent was used to isolate total RNA following the manufacturer's protocol. Briefly, RNA samples underwent denaturation at 95°C for 3 minutes, followed by immediate chilling on ice. An Amersham Hybond-N$^+$ membrane was utilized to load quantified RNA samples. After UV cross-linking, 5% defatted skim milk in PBST buffer was employed to block the membrane for 1 hour. Subsequently, it was incubated with anti-m^{6}A antibodies (68055-1-Ig, Proteintech) for 2 hours, while staining of the loading control with 0.02% methylene blue in 0.3 M sodium acetate at room temperature was performed. After three washes with PBST, the experimental membrane was exposed to HRP-conjugated goat anti-rabbit IgG for 1 hour at room temperature and then subjected to detection using Pierce ECL Plus Western Blotting Substrate (Thermo Fisher Scientific, Waltham, USA).

DNA dot blot

The TIANamp Genomic DNA Kit (Cat: DP304, TIANGEN Biotech) was used to isolate total DNA according to the manufacturer's protocol. Briefly, DNA samples were diluted with 0.1 M NAOH and denatured at 95°C for 10 minutes, after which they were immediately put on ice. The remaining steps were conducted similarly to the m^{6}A dot blot assay, but with the use of an anti-5-mC antibody (28692, Cell Signaling Technology).

Animal Experiments

GemPharmatech (Nanjing, China) provided 6–8-week-old female C57BL/6 mice for the experiments. The animals were randomly assigned to groups and the investigator was blinded to the group allocation before the experimental procedures were performed. In the *in vivo* immune cell depletion mouse model, InVivoMAb anti-mouse CD8β (BE0223, BioXcell) was used to deplete $CD8^+$ T cells in mice. In the subcutaneous tumor model and immunotherapy model, 5×10^5 MB49-shNC/MB49-shYTHDF1 cells were injected subcutaneously into the right flank of each mouse. When tumors were palpable, each mouse received intraperitoneal injections of 100 μg anti-mouse PD-L1 (BE0101, BioXcell) antibody or IgG2a (BE0085, BioXcell) isotype control antibody every three days. Tumor volume was measured every two days using calipers or observed using the IVIS system (PerkinElmer, USA).

In the orthotopic bladder tumor model, 5×10^6 MB49-luci cells were intravesically irrigated into the bladder after voiding. For the bladder irrigation model, VNP20009 bacteria were resuspended in PBS to a final concentration of 1×10^8 CFU (colony forming unit)/mL, with PBS alone as control. Mice were anesthetized and a 24-gauge catheter was inserted into the bladder through the urethra, and 50 μL of VNP20009 (purchased from BIOSCI company, Hangzhou) or PBS was instilled into the bladder. Mice were euthanized at the end of the experiment or when reaching the ethical endpoint for animal welfare. All animal experiments were approved by the Institutional Animal Care and Use Committee of Sun Yat-sen University.

Single-cell RNA sequencing

Single-cell RNA sequencing of the tumors was performed using the 10x Genomics platform as follows: Fresh mouse tumor tissues were collected 30 days post-inoculation. Single-cell suspensions were prepared by mechanical disruption followed by enzymatic digestion with Type IV Collagenase (C5138, Sigma) and hyaluronidase (HX0514, Sigma). The suspensions were immediately processed for single-cell RNA sequencing (scRNA-seq). The resulting data were processed, and libraries were constructed according to the manufacturer's protocol.

Flow cytometry

Tumor samples were collected and digested into single-cell suspensions. Subsequently, tumor-infiltrating lymphocytes (TILs) were extracted using Lymphoprep™ (07801, STEMCELL) through gradient centrifugation. After washing twice with PBS, the cells were used for subsequent experiments. For cell surface marker analysis, samples were first stained with Zombie Aqua dye (423102, Biolegend) at room temperature for 15 minutes before staining with different antibodies. The cells were resuspended and stained with specified antibodies at 4°C for 30 minutes. Intracellular fixation and permeabilization were performed using the buffer set from Invitrogen. For the detection of intracellular effector molecules and cytokines, T cells were treated with RPMI-1640 medium containing ionomycin (M3621, AbMole), PMA (M4647, AbMole), and GolgiStop (M6991, AbMole) for 4 hours. The Stained cells were detected using a BD LSRFortessa, and data analysis was conducted with FlowJo software (version 10.8.1). The antibodies were used as follows: anti-mouse CD45 (30-F11, BioLegend), anti-mouse CD8 (53-6.7, BioLegend), anti-mouse CD4 (GK1.5, Biolegend), anti-mouse CD44 (IM7, Biolegend), anti-mouse CD62L (MEL-14, Biolegend), anti-mouse IFN-γ (XMG1.2, BioLegend) , anti-mouse TNF-α (MP6-XT22, BioLegend), anti-mouse PD-L1 (10F.9G2, BioLegend), anti-mouse Slamf6 (330-AJ, BioLegend), anti-mouse Cxcr6(SA051D1, BioLegend), anti-mouse MHC-I (AF6-88.5, Biolegend), anti-human PD-L1 (29E.2A3, BioLegend) and anti-human HLA-A (W6/32, BioLegend).

TissueFAXS panoramic analyses

To assess the relationship between YTHDF1$^+$ cells, PD-L1$^+$ cells, RIG-1$^+$ cells, and CD8$^+$ T cells, we utilized TissueFAXS panoramic analysis (Tissue Gnostics, Austria) to detect the expression of target cells and molecules stained with multiple immunofluorescent markers in bladder cancer patients responding to immunotherapy and those resistant to it. In brief, tissue samples were prepared and fixed using formalin, followed by embedding in paraffin and sectioning at a thickness of 4-5 micrometers. The sections were stained using immunofluorescence with the TSA (tyramide signal amplification) method. High-resolution panoramic images of the stained sections were obtained using the TissueFAXS system. The images were analyzed with TissueFAXS software to quantify cellular and structural features.

Immunohistochemistry

Briefly, paraffin-embedded tissues were deparaffinized, rehydrated, subjected to antigen retrieval, blocked for peroxidases, blocked again, incubated with antibodies, and stained. All reagents were from ZSGB-BIO. Scoring was independently conducted by two experienced pathologists.

Statistical analyses

To compare parameters between the two groups, independent sample t-tests or Mann-Whitney U tests were employed for continuous variables, while chi-squared tests or Fisher's exact tests were used for dichotomous variables. Correlations between variables were assessed using Pearson or Spearman correlation coefficients. Kaplan-Meier survival curves illustrated prognostic analyses of dichotomous variables, with significance evaluated by the log-rank test. The similarity of variance between the groups being statistically compared was assessed. Data analyses were conducted using GraphPad Prism 8 (GraphPad Software) and R software (version 4.0). P-values below 0.05 were considered statistically significant.

Figure S1-related to Figure 1. Effects of RIG-I and MAVS expression, mRNA stability, and m^6A modification on IFN-β production in 5637 and T24 cells under methionine restriction and IFN-γ stimulation. (A) IFN-β production levels in 5637 cells under various conditions. (B) mRNA stability analysis of MAVS mRNA in 5637 cells under MFM or NM conditions following Actinomycin D treatment. (C) RIP analysis showing the relative expression of MAVS mRNA in 5637 cells with IgG or m^6A antibody under MFM and NM conditions. (D) IFN-β production in 5637 cells under different conditions. (E) IFN-β production in T24 (left) and 5637 (right) cells under different treatment conditions. ns, P>0.05, *P<0.05, **P<0.01, ***P<0.001, ****P<0.0001. NM: Normal Medium, MFM: Methionine Free Medium, RIP: RNA immunoprecipitation, TFMT: Trifluoromethyl-tubercidin.

Figure S2-related to Figure 1. The impact of IFNAR1 on functional changes induced by methionine restriction. (A) IFN-β production levels in 5637 cells under various conditions. (B) LDH cytotoxicity assay detecting the percentage of cytotoxicity after co-culturing T24-shNC/T24-shIFNAR1 cells with activated human PBMCs under NM and MFM conditions. (C-E) Tumor growth (C), images of tumors (D), and tumor weights (E) of MB49-shNC/MB49-shIFNAR1 in mice under CD and MRD feeding conditions. (F) Representative flow cytometry plots and bar graphs displaying the proportions of tumor-infiltrating $CD4^+$ and $CD8^+$ T cells per gram of tumor tissue in each treatment group. (G) Flow cytometry plots and bar graphs illustrating the proportions of $CD8^+$ T cells producing IFN-γ and TNF-α in each treatment group. ns, P>0.05, *P<0.05, **P<0.01, ***P<0.001, ****P<0.0001. NM: Normal Medium, MFM: Methionine Free Medium, CD: Complete Diet, MRD: Methionine Restricted Diet.

Figure S3-related to Figure 2. Methionine regulates the expression of YTHDF1. (A) RNA degradation assays showed YTHDF1 RNA degradation following NM or MFM treatment in T24 and 5637 cells. (B) Schematic diagram of methionine cycle. (C-D) Western blot and RT-qPCR were used to detect YTHDF1 expression in T24 (C) and 5637 (D) cells following treatment with methionine (Met), S-adenosylmethionine (SAM), S-adenosylhomocysteine (SAH), and homocysteine (Homo-cys) at various time points. *P<0.05, **P<0.01, ***P<0.001.

Figure S4-related to Figure 2. DDX58 is positively associated with immune infiltration in the TCGA-BLCA database. (A) Heatmap of the relationship between DDX58 expression and therapeutic targets. (B) Correlation between DDX58 expression and different recruited immune cells. (C) Correlation between DDX58 expression and different infiltrated immune cells. (D) Heatmap of the relationship between DDX58 expression and molecular subtypes of bladder cancer. *P<0.05, **P<0.01, ***P<0.001.

Figure S5-related to Figure 2. DDX58 is positively associated with interferon activation and survival in bladder cancer. (A) The enrichment pathways between different DDX58 expression. (B) The survival probability of CD8 expression in TCGA-BLCA. (C) The survival probability of IFN expression in TCGA-BLCA. (D) Heatmap shows the expression of YTHDF1 and interferon-stimulated genes in T24 cells under NM and MFM conditions detected by RNA-seq. (E-F) RT-qPCR detecting the differences in YTHDF1, ISG15, MX1, IFI6 and ISG20 mRNA expression in 5637 (E) and T24 (F) cells between the negative control and YTHDF1 knockdown groups. **P<0.01, ***P<0.001, ****P<0.0001.

Figure S6-related to Figure 4. YTHDF1 expression is negatively associated with HLA expression in bladder cancer. (A) Represent Histogram and bar graph show the expression of HLA-A in shNC and shYTHDF1 T24 cells. (B) Represent Histogram and bar graph show the expression of HLA-A in shNC, shYTHDF1, shYTHDF1&shIFNAR1, shYTHDF1&shMAVS and shYTHDF1&shRIG-I T24 cells. (C) Gating strategies for flow cytometry in $CD4^+$ and $CD8^+$ T cells. (D) FMO plots for intracellular staining and gating strategies. *P<0.05, **P<0.01, ****P<0.0001.

Figure S7-related to Figure 5. Methionine restriction promotes immune cell infiltration in bladder cancer and synergizes with immune checkpoint inhibitors. (A) Infiltration status of different cell types among various treated groups. (B) UMAP plot of different macrophage subpopulations. (C) UMAP plot of macrophage subpopulations in different experimental treatment groups. (D) Enrichment analysis plot of differentially expressed genes in macrophages across different treatment groups. **P<0.01, ****P<0.0001.

Figure S8-related to Figure 7. YTHDF1 interacts with eIF5B and promotes the translation of PD-L1. (A) Representative immunofluorescence images of tumor tissues from T24 cells showing the correlation between endogenous YTHDF1 and eIF5B. (B) Western Blot analysis

showing the expression of eIF5B and PD-L1 proteins in T24 cell line under conditions with or without IFN-γ and methionine treatment (upper) and the expression of eIF5B and PD-L1 proteins in T24 cell line under conditions with or without IFN-γ and shYTHDF1(bottom). (C)Western Blot analysis showing the expression of YTHDF1, eIF5B, and PD-L1 proteins after over expression of YTHDF1 or not in T24 and 5637 cells. (D) Western Blot analysis showing the expression of eIF5B and PD-L1 proteins in different treated groups. (E)Western blot analysis of YTHDF1, eIF5B, and PD-L1 in T24 and 5637 cells treated with IFN-γ, with or without MFM and NM, under shIFNAR1 or control conditions.

Figure S9. Schematic diagram of the study.

Table S1. Sequence of shRNAs, sgRNAs and siRNAs.

Table S2. Primer Sequences used in RT-qPCR.

Table S3. RNA-seq results.

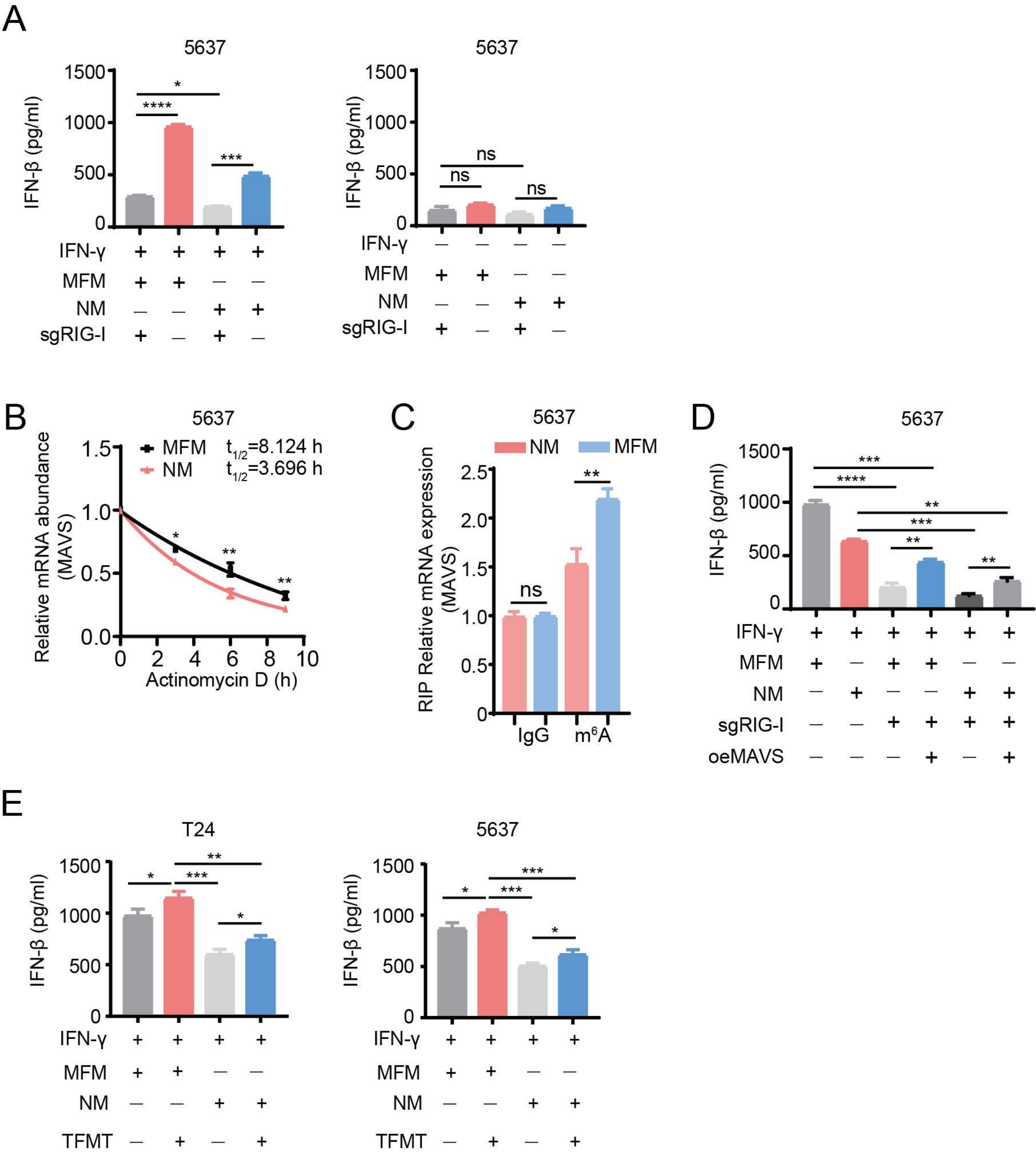

A
5637
IFN-β (pg/ml)
IFN-γ
MFM
NM
sgRIG-I
B
5637
MFM t1/2=8.124 h
NM t1/2=3.696 h
Relative mRNA abundance (MAVS)
Actinomycin D (h)
C
5637
NM
MFM
RIP Relative mRNA expression (MAVS)
IgG
m6A
D
5637
oeMAVS
E
T24
5637
TFMT

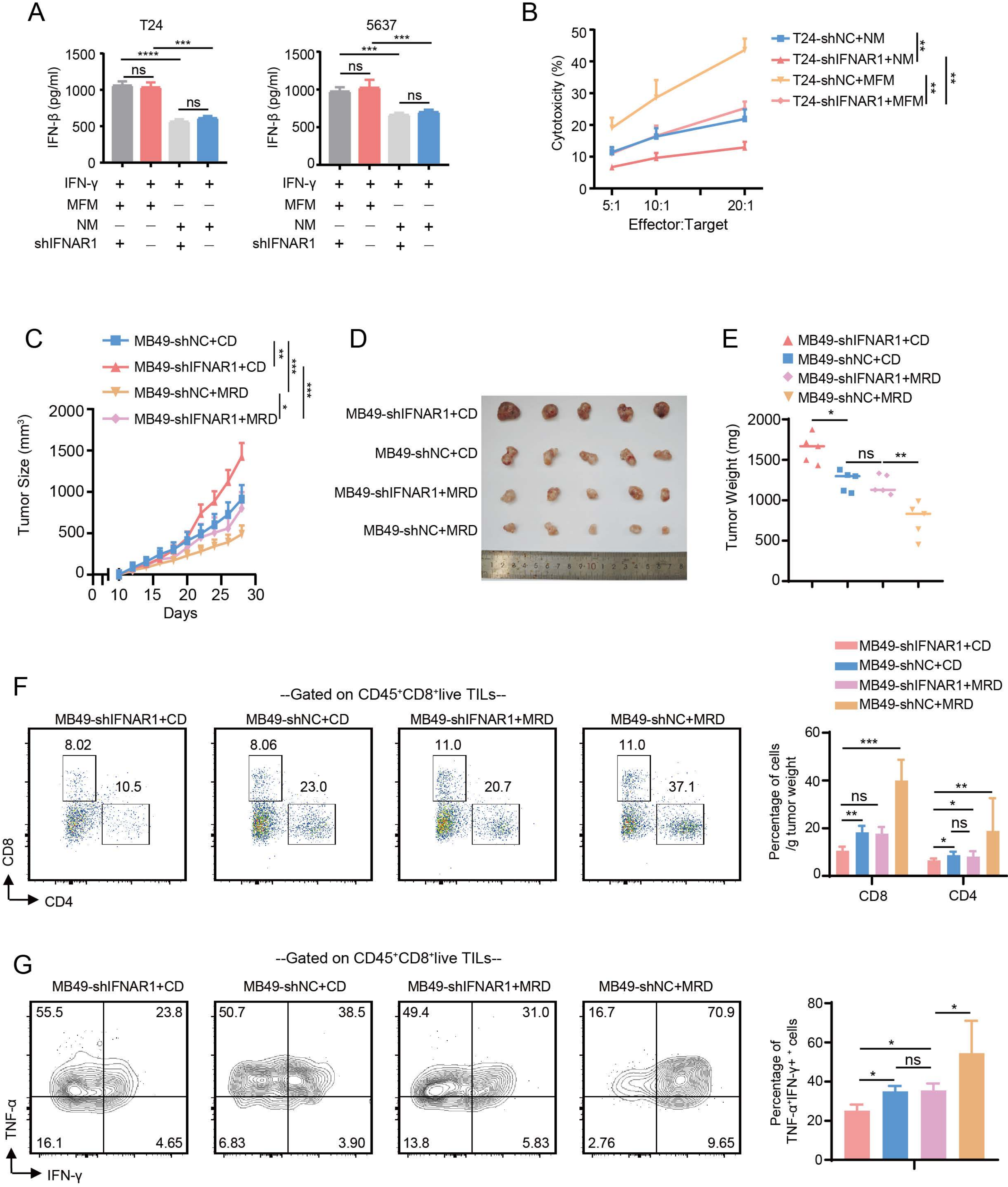
A
T24
5637
IFN-β (pg/ml)
IFN-γ
MFM
NM
shIFNAR1
ns
****
***
B
Cytotoxicity (%)
Effector:Target
5:1
10:1
20:1
T24-shNC+NM
T24-shIFNAR1+NM
T24-shNC+MFM
T24-shIFNAR1+MFM
C
MB49-shNC+CD
MB49-shIFNAR1+CD
MB49-shNC+MRD
MB49-shIFNAR1+MRD
Tumor Size (mm³)
Days
D
MB49-shIFNAR1+CD
MB49-shNC+CD
MB49-shIFNAR1+MRD
MB49-shNC+MRD
E
MB49-shIFNAR1+CD
MB49-shNC+CD
MB49-shIFNAR1+MRD
MB49-shNC+MRD
Tumor Weight (mg)
F
--Gated on CD45⁺CD8⁺live TILs--
MB49-shIFNAR1+CD
MB49-shNC+CD
MB49-shIFNAR1+MRD
MB49-shNC+MRD
8.02
10.5
8.06
23.0
11.0
20.7
11.0
37.1
CD8
CD4
MB49-shIFNAR1+CD
MB49-shNC+CD
MB49-shIFNAR1+MRD
MB49-shNC+MRD
Percentage of cells /g tumor weight
CD8
CD4
G
--Gated on CD45⁺CD8⁺live TILs--
MB49-shIFNAR1+CD
MB49-shNC+CD
MB49-shIFNAR1+MRD
MB49-shNC+MRD
55.5
23.8
16.1
4.65
50.7
38.5
6.83
3.90
49.4
31.0
13.8
5.83
16.7
70.9
2.76
9.65
TNF-α
IFN-γ
Percentage of TNF-α⁺IFN-γ⁺ cells

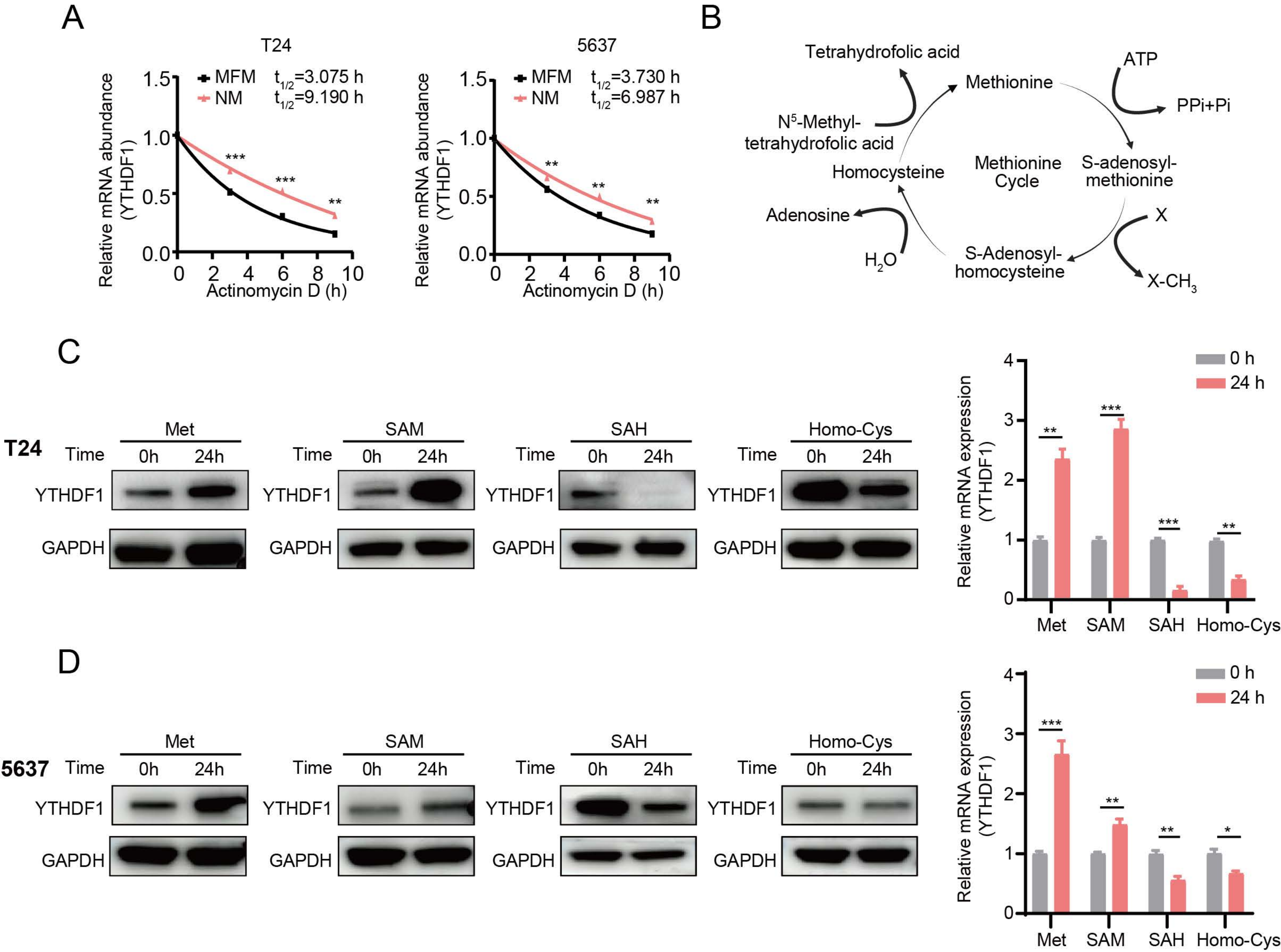

A
T24
MFM $t_{1/2}$=3.075 h
NM $t_{1/2}$=9.190 h
Relative mRNA abundance (YTHDF1)
Actinomycin D (h)
5637
MFM $t_{1/2}$=3.730 h
NM $t_{1/2}$=6.987 h
B
Tetrahydrofolic acid
Methionine
ATP
PPi+Pi
N^5-Methyl-tetrahydrofolic acid
Homocysteine
Methionine Cycle
S-adenosyl-methionine
Adenosine
X
H_2O
S-Adenosyl-homocysteine
X-CH_3
C
T24
Met
SAM
SAH
Homo-Cys
Time
0h
24h
YTHDF1
GAPDH
Relative mRNA expression (YTHDF1)
0 h
24 h
D
5637

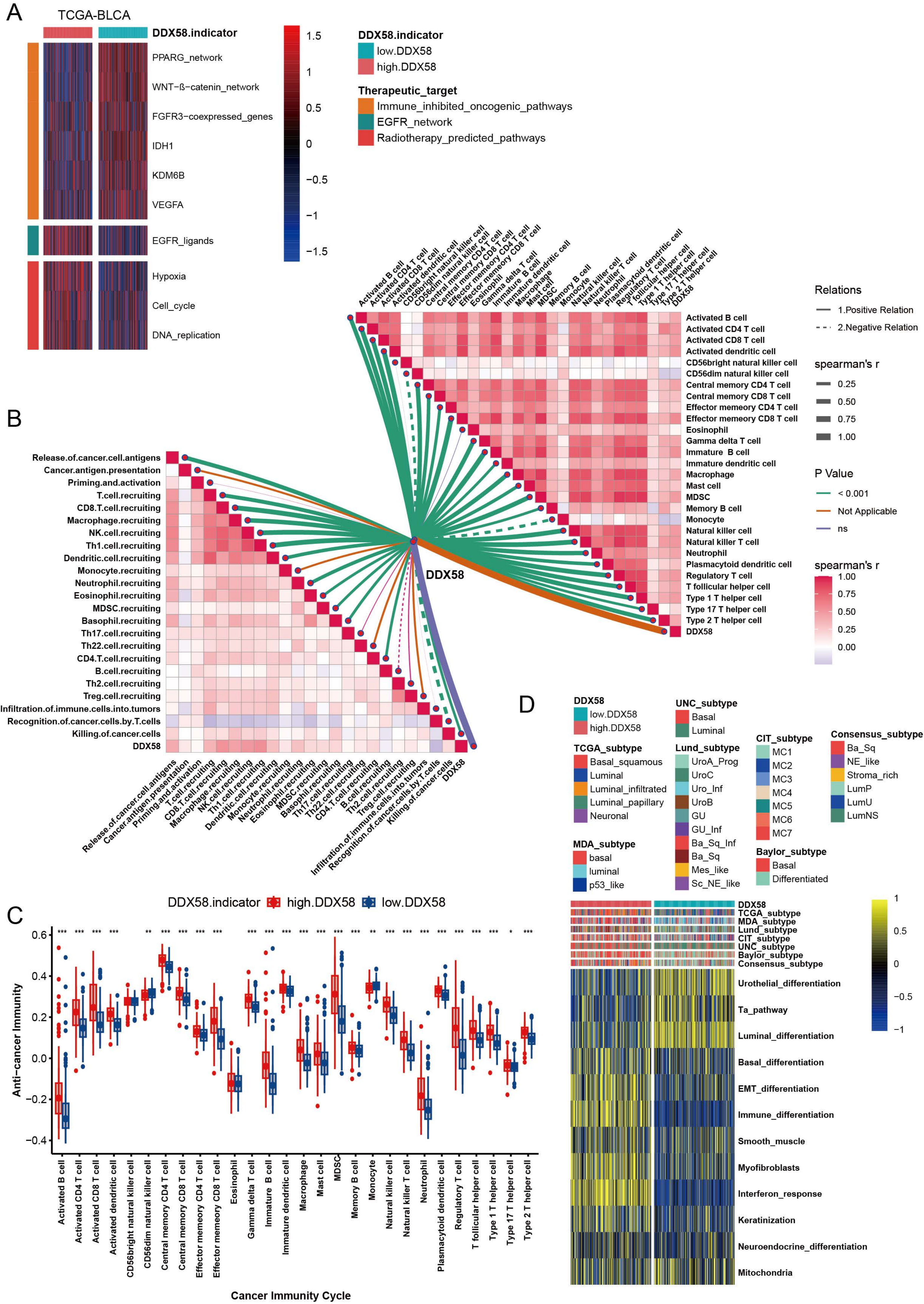

A
TCGA-BLCA
DDX58.indicator
PPARG_network
WNT−ß−catenin_network
FGFR3−coexpressed_genes
IDH1
KDM6B
VEGFA
EGFR_ligands
Hypoxia
Cell_cycle
DNA_replication
1.5
1
0.5
0
−0.5
−1
−1.5
DDX58.indicator
low.DDX58
high.DDX58
Therapeutic_target
Immune_inhibited_oncogenic_pathways
EGFR_network
Radiotherapy_predicted_pathways
Activated B cell
Activated CD4 T cell
Activated CD8 T cell
Activated dendritic cell
CD56bright natural killer cell
CD56dim natural killer cell
Central memory CD4 T cell
Central memory CD8 T cell
Effector memeory CD4 T cell
Effector memeory CD8 T cell
Eosinophil
Gamma delta T cell
Immature B cell
Immature dendritic cell
Macrophage
Mast cell
MDSC
Memory B cell
Monocyte
Natural killer cell
Natural killer T cell
Neutrophil
Plasmacytoid dendritic cell
Regulatory T cell
T follicular helper cell
Type 1 T helper cell
Type 17 T helper cell
Type 2 T helper cell
DDX58
Relations
1.Positive Relation
2.Negative Relation
spearman's r
0.25
0.50
0.75
1.00
P Value
< 0.001
Not Applicable
ns
spearman's r
1.00
0.75
0.50
0.25
0.00
B
Release.of.cancer.cell.antigens
Cancer.antigen.presentation
Priming.and.activation
T.cell.recruiting
CD8.T.cell.recruiting
Macrophage.recruiting
NK.cell.recruiting
Th1.cell.recruiting
Dendritic.cell.recruiting
Monocyte.recruiting
Neutrophil.recruiting
Eosinophil.recruiting
MDSC.recruiting
Basophil.recruiting
Th17.cell.recruiting
Th22.cell.recruiting
CD4.T.cell.recruiting
B.cell.recruiting
Th2.cell.recruiting
Treg.cell.recruiting
Infiltration.of.immune.cells.into.tumors
Recognition.of.cancer.cells.by.T.cells
Killing.of.cancer.cells
DDX58
DDX58
C
DDX58.indicator
high.DDX58
low.DDX58
Anti−cancer Immunity
0.6
0.4
0.2
0.0
−0.2
−0.4
*** *** *** *** ** *** *** *** *** *** *** *** *** *** *** *** ** *** *** *** *** *** *** *** * ***
Activated B cell
Activated CD4 T cell
Activated CD8 T cell
Activated dendritic cell
CD56bright natural killer cell
CD56dim natural killer cell
Central memory CD4 T cell
Central memory CD8 T cell
Effector memeory CD4 T cell
Effector memeory CD8 T cell
Eosinophil
Gamma delta T cell
Immature B cell
Immature dendritic cell
Macrophage
Mast cell
MDSC
Memory B cell
Monocyte
Natural killer cell
Natural killer T cell
Neutrophil
Plasmacytoid dendritic cell
Regulatory T cell
T follicular helper cell
Type 1 T helper cell
Type 17 T helper cell
Type 2 T helper cell
Cancer Immunity Cycle
D
DDX58
low.DDX58
high.DDX58
UNC_subtype
Basal
Luminal
TCGA_subtype
Basal_squamous
Luminal
Luminal_infiltrated
Luminal_papillary
Neuronal
Lund_subtype
UroA_Prog
UroC
Uro_Inf
UroB
GU
GU_Inf
Ba_Sq_Inf
Ba_Sq
Mes_like
Sc_NE_like
CIT_subtype
MC1
MC2
MC3
MC4
MC5
MC6
MC7
Consensus_subtype
Ba_Sq
NE_like
Stroma_rich
LumP
LumU
LumNS
MDA_subtype
basal
luminal
p53_like
Baylor_subtype
Basal
Differentiated
DDX58
TCGA_subtype
MDA_subtype
Lund_subtype
CIT_subtype
UNC_subtype
Baylor_subtype
Consensus_subtype
Urothelial_differentiation
Ta_pathway
Luminal_differentiation
Basal_differentiation
EMT_differentiation
Immune_differentiation
Smooth_muscle
Myofibroblasts
Interferon_response
Keratinization
Neuroendocrine_differentiation
Mitochondria
1
0.5
0
−0.5
−1

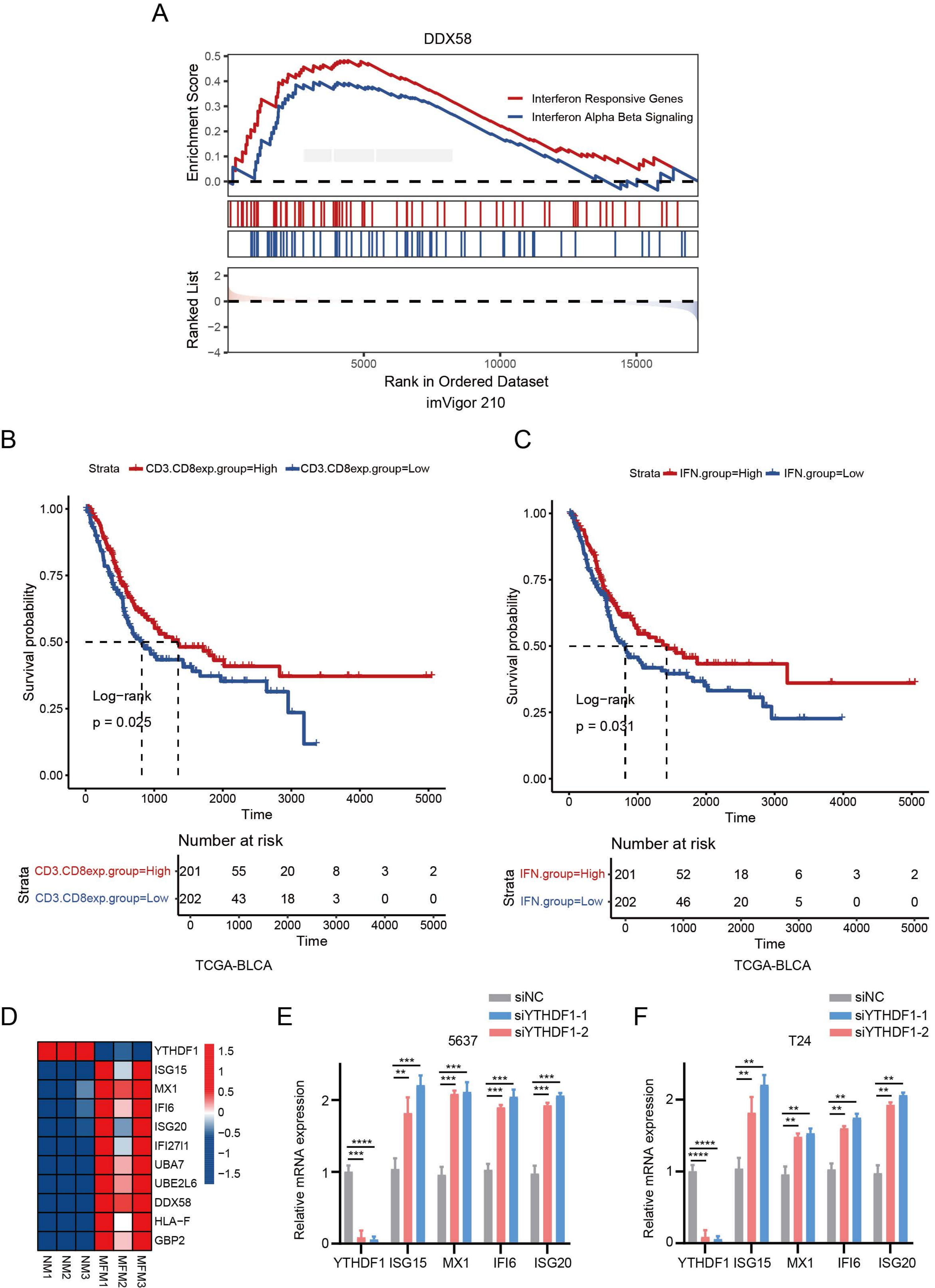

A
DDX58
Enrichment Score
Interferon Responsive Genes
Interferon Alpha Beta Signaling
Ranked List
Rank in Ordered Dataset
imVigor 210
B
Strata CD3.CD8exp.group=High CD3.CD8exp.group=Low
Survival probability
Log−rank
p = 0.025
Time
Number at risk
Strata
CD3.CD8exp.group=High 201 55 20 8 3 2
CD3.CD8exp.group=Low 202 43 18 3 0 0
TCGA-BLCA
C
Strata IFN.group=High IFN.group=Low
Survival probability
Log−rank
p = 0.031
Time
Number at risk
Strata
IFN.group=High 201 52 18 6 3 2
IFN.group=Low 202 46 20 5 0 0
TCGA-BLCA
D
YTHDF1
ISG15
MX1
IFI6
ISG20
IFI27l1
UBA7
UBE2L6
DDX58
HLA−F
GBP2
NM1 NM2 NM3 MFM1 MFM2 MFM3
E
siNC
siYTHDF1-1
siYTHDF1-2
5637
Relative mRNA expression
YTHDF1 ISG15 MX1 IFI6 ISG20
F
siNC
siYTHDF1-1
siYTHDF1-2
T24
Relative mRNA expression
YTHDF1 ISG15 MX1 IFI6 ISG20

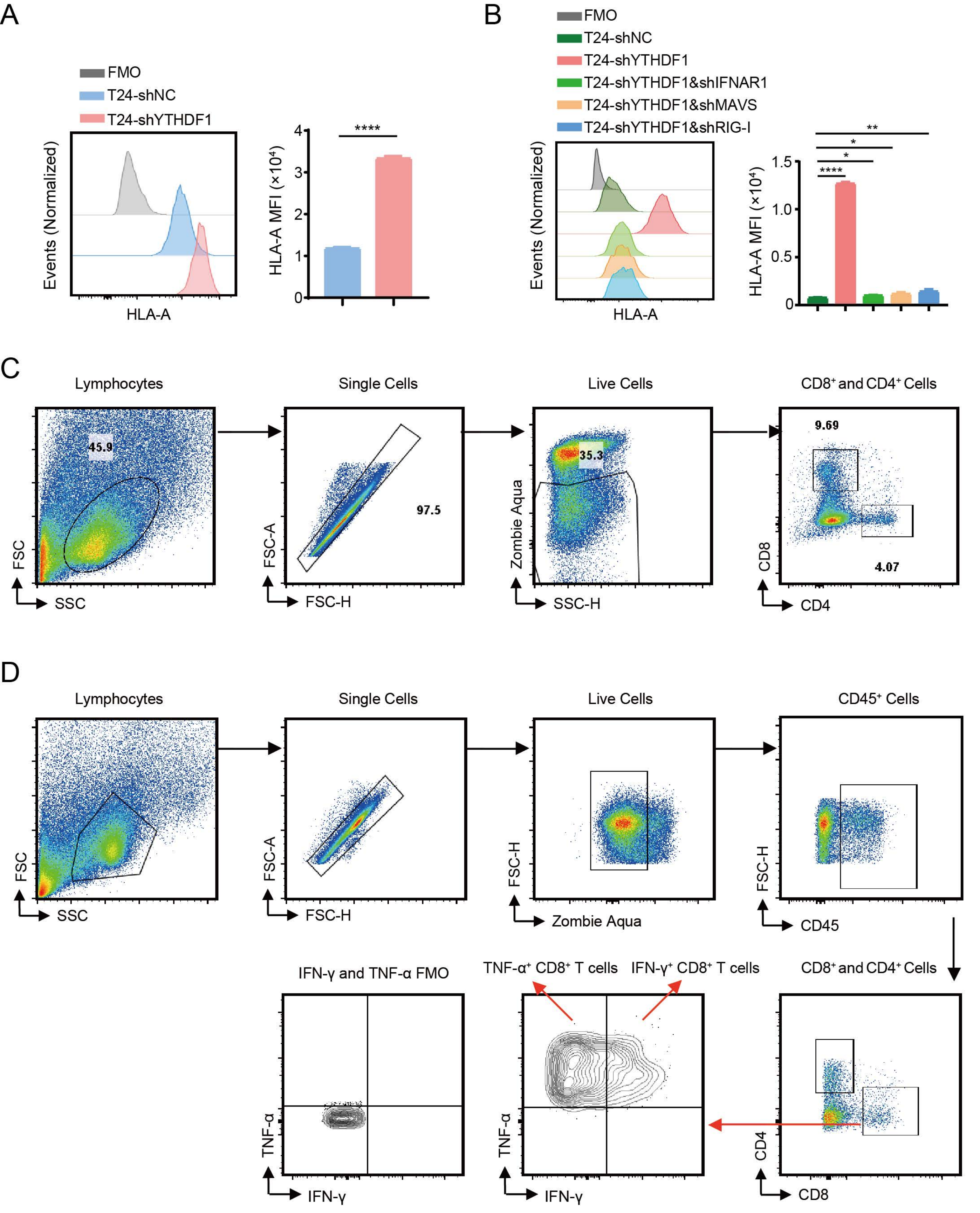

A
FMO
T24-shNC
T24-shYTHDF1
Events (Normalized)
HLA-A
HLA-A MFI (×10⁴)
****
B
FMO
T24-shNC
T24-shYTHDF1
T24-shYTHDF1&shIFNAR1
T24-shYTHDF1&shMAVS
T24-shYTHDF1&shRIG-I
Events (Normalized)
HLA-A
HLA-A MFI (×10⁴)
**
*
*
****
C
Lymphocytes
Single Cells
Live Cells
CD8⁺ and CD4⁺ Cells
45.9
97.5
35.3
9.69
4.07
FSC
SSC
FSC-A
FSC-H
Zombie Aqua
SSC-H
CD8
CD4
D
Lymphocytes
Single Cells
Live Cells
CD45⁺ Cells
FSC
SSC
FSC-A
FSC-H
FSC-H
Zombie Aqua
FSC-H
CD45
IFN-γ and TNF-α FMO
TNF-α⁺ CD8⁺ T cells
IFN-γ⁺ CD8⁺ T cells
CD8⁺ and CD4⁺ Cells
TNF-α
IFN-γ
TNF-α
IFN-γ
CD4
CD8

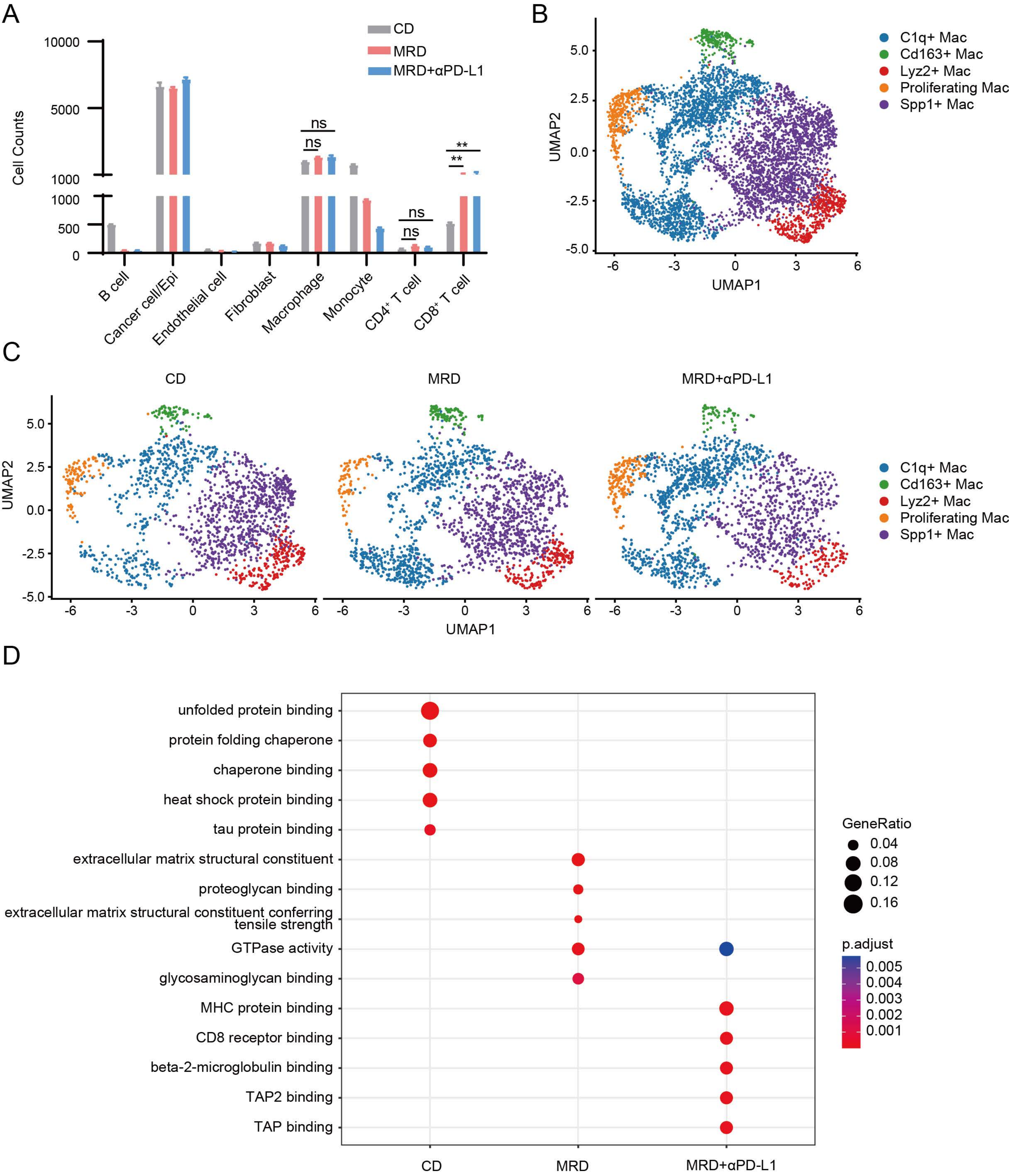
A
CD
MRD
MRD+αPD-L1
Cell Counts
10000
5000
1000
1000
500
0
ns
ns
ns
ns
**
**
B cell
Cancer cell/Epi
Endothelial cell
Fibroblast
Macrophage
Monocyte
CD4+ T cell
CD8+ T cell
B
UMAP2
UMAP1
C1q+ Mac
Cd163+ Mac
Lyz2+ Mac
Proliferating Mac
Spp1+ Mac
C
CD
MRD
MRD+αPD-L1
UMAP2
UMAP1
C1q+ Mac
Cd163+ Mac
Lyz2+ Mac
Proliferating Mac
Spp1+ Mac
D
unfolded protein binding
protein folding chaperone
chaperone binding
heat shock protein binding
tau protein binding
extracellular matrix structural constituent
proteoglycan binding
extracellular matrix structural constituent conferring tensile strength
GTPase activity
glycosaminoglycan binding
MHC protein binding
CD8 receptor binding
beta-2-microglobulin binding
TAP2 binding
TAP binding
CD
MRD
MRD+αPD-L1
GeneRatio
0.04
0.08
0.12
0.16
p.adjust
0.005
0.004
0.003
0.002
0.001

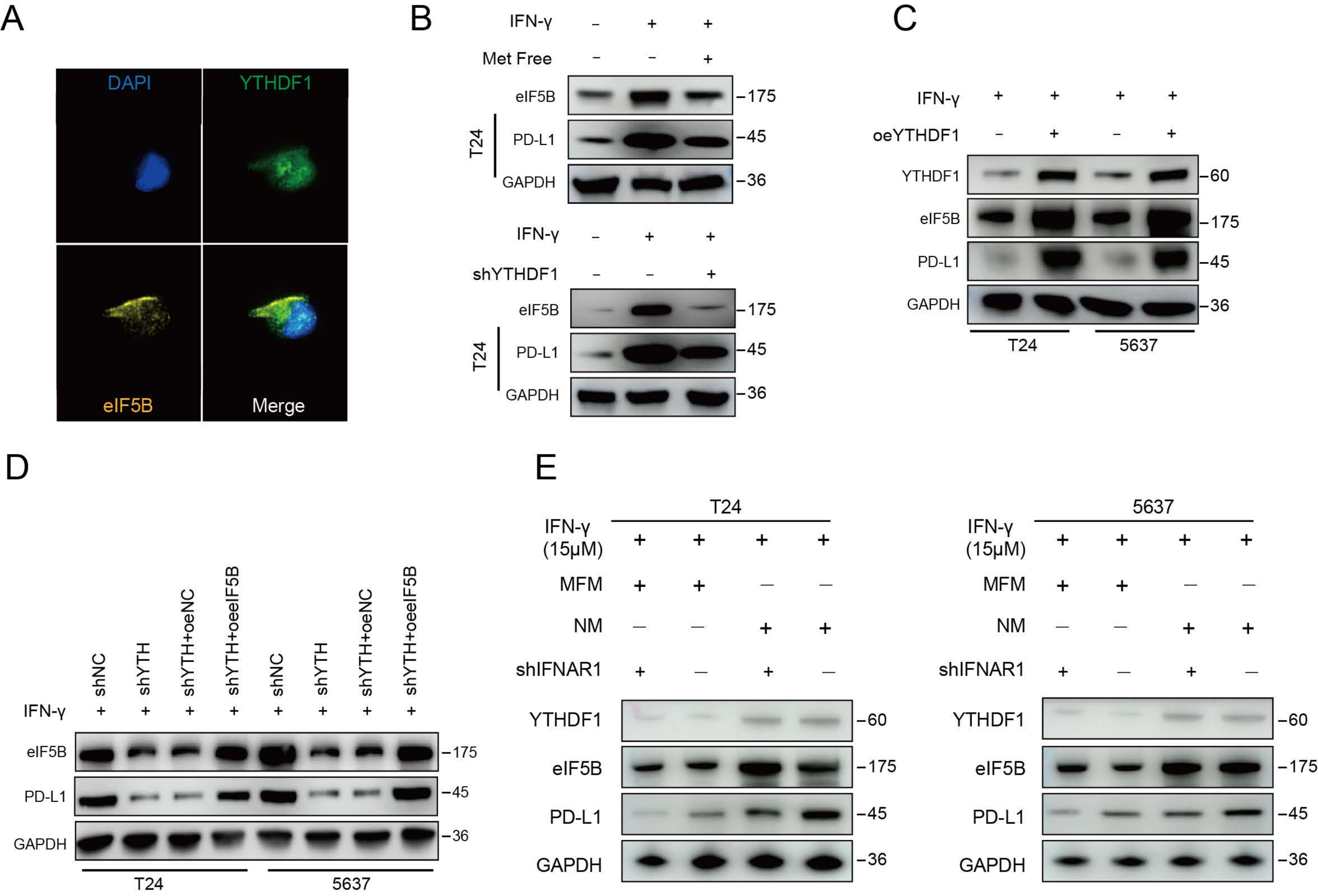

A
DAPI
YTHDF1
eIF5B
Merge
B
IFN-γ – + +
Met Free – – +
eIF5B
PD-L1
GAPDH
T24
–175
–45
–36
IFN-γ – + +
shYTHDF1 – – +
eIF5B
PD-L1
GAPDH
T24
–175
–45
–36
C
IFN-γ + + + +
oeYTHDF1 – + – +
YTHDF1
eIF5B
PD-L1
GAPDH
–60
–175
–45
–36
T24
5637
D
shNC
shYTH
shYTH+oeNC
shYTH+oeeIF5B
shNC
shYTH
shYTH+oeNC
shYTH+oeeIF5B
IFN-γ + + + + + + + +
eIF5B
PD-L1
GAPDH
–175
–45
–36
T24
5637
E
T24
IFN-γ (15μM) + + + +
MFM + + — —
NM — — + +
shIFNAR1 + — + —
YTHDF1
eIF5B
PD-L1
GAPDH
–60
–175
–45
–36
5637
IFN-γ (15μM) + + + +
MFM + + — —
NM — — + +
shIFNAR1 + — + —
YTHDF1
eIF5B
PD-L1
GAPDH
–60
–175
–45
–36
