# Supplementary material for: Methionine-driven YTHDF1 expression facilitates bladder cancer progression by attenuating RIG-I-modulated immune responses and enhancing the eIF5B-PD-L1 axis
Source: Cell Death Differ. 2024 Dec 13;32(4):776–91. doi: 10.1038/s41418-024-01434-y (PMC11982326; doi:10.1038/s41418-024-01434-y)

Figure 1D

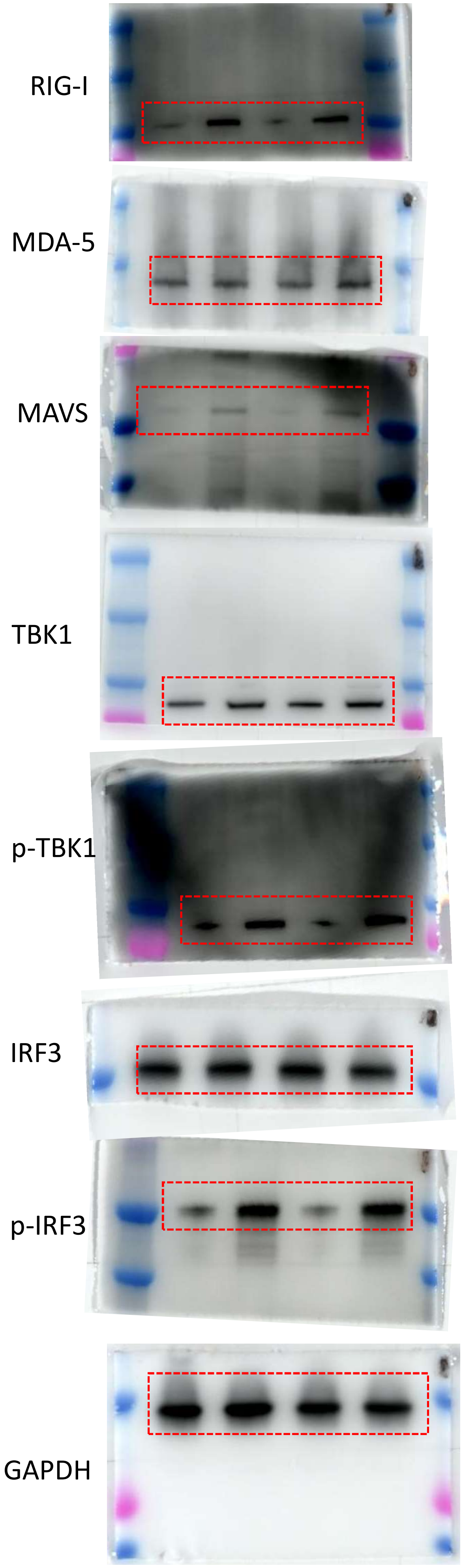

Figure 1F

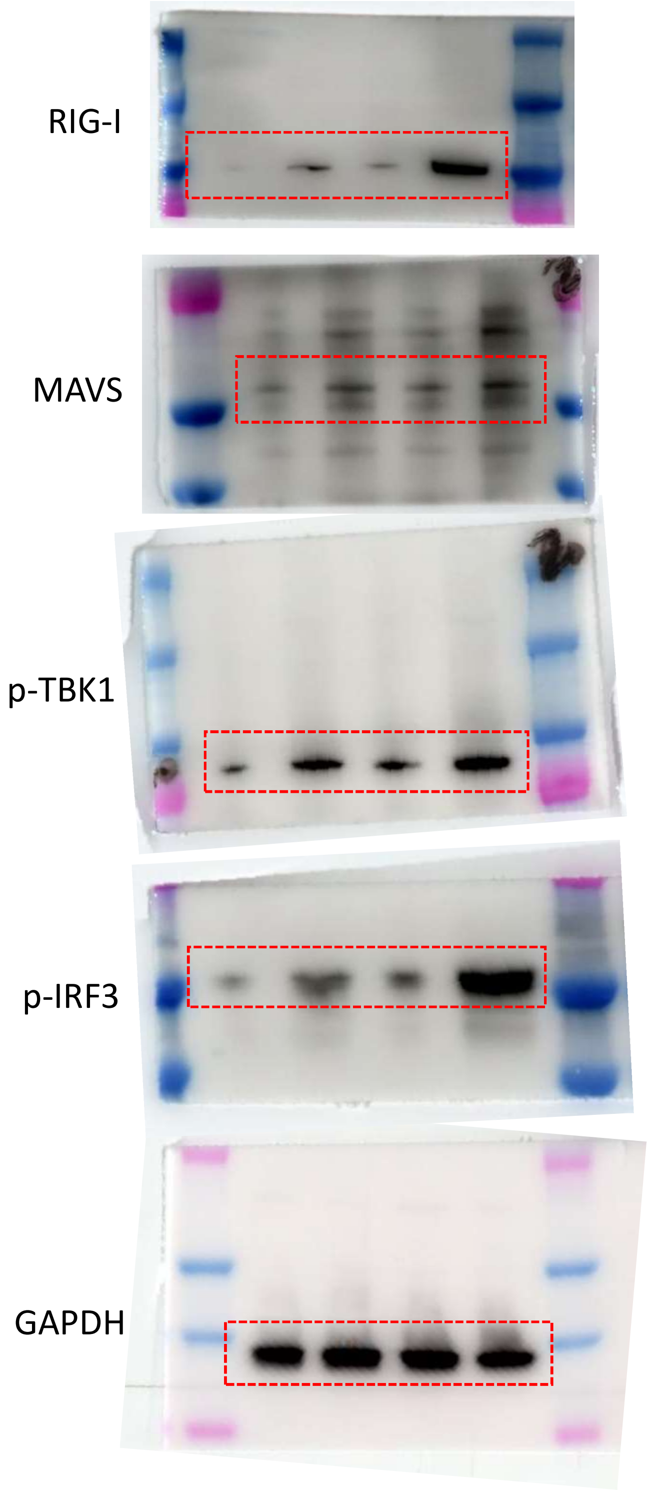

Figure 1H

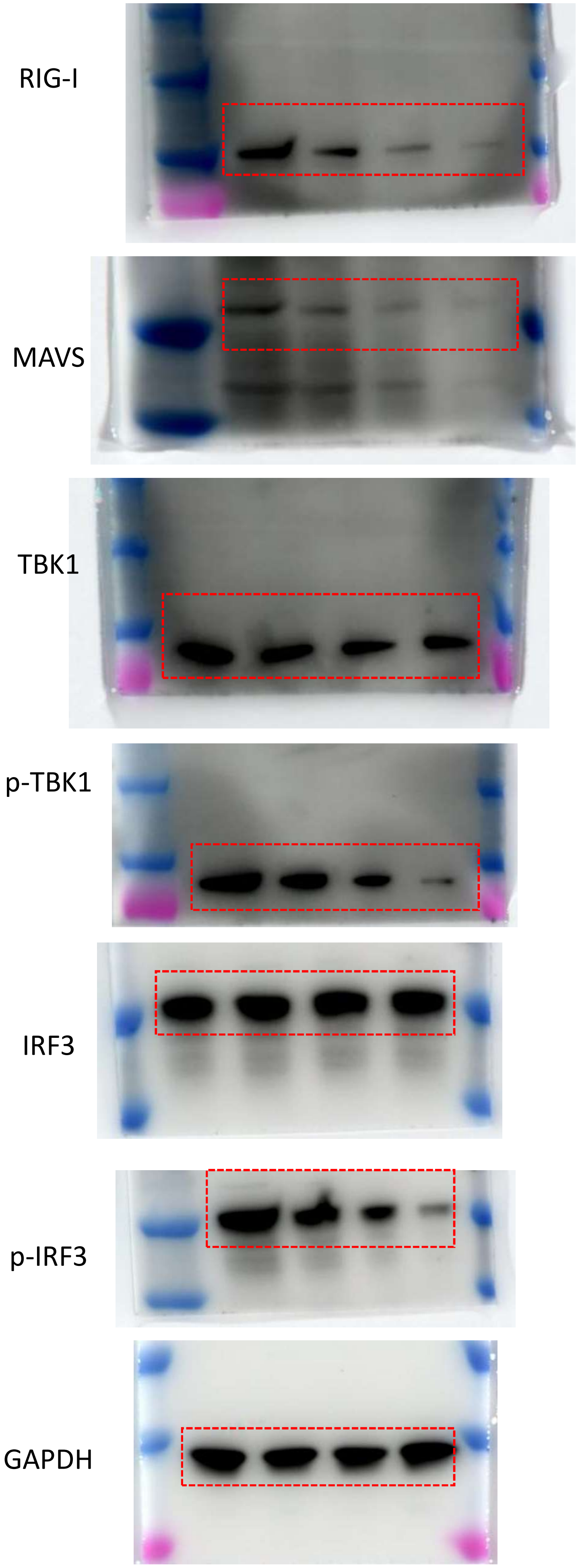

Figure 1J

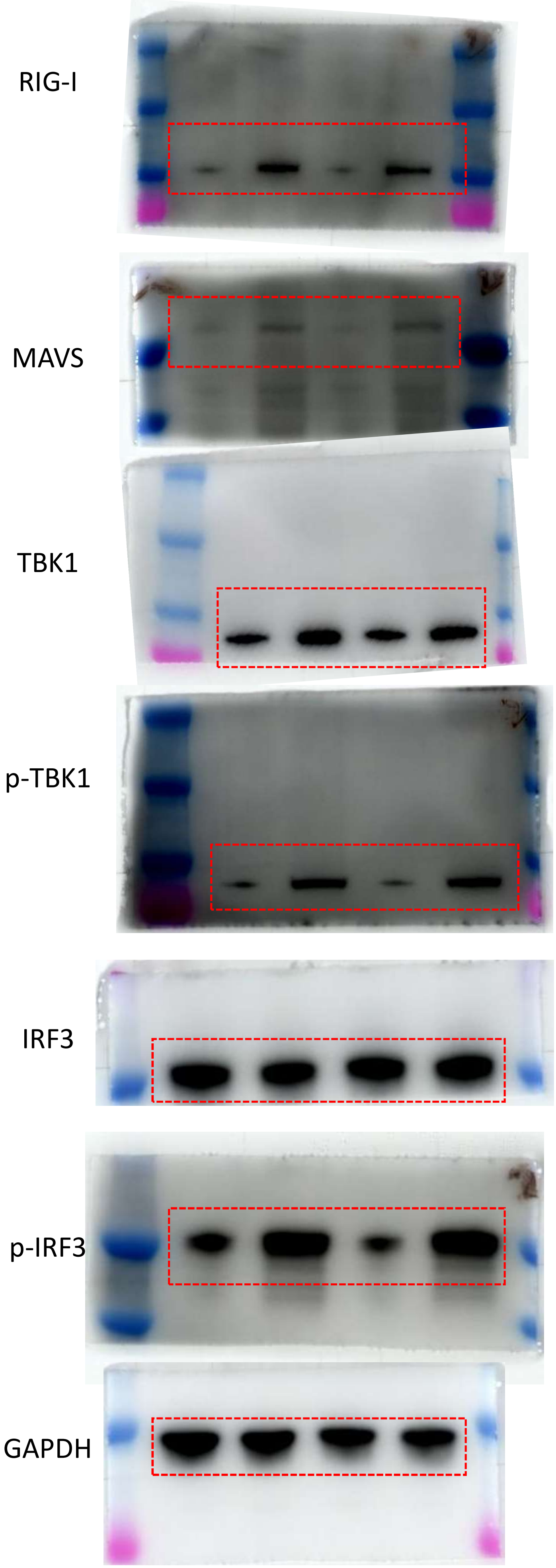

Figure 1P

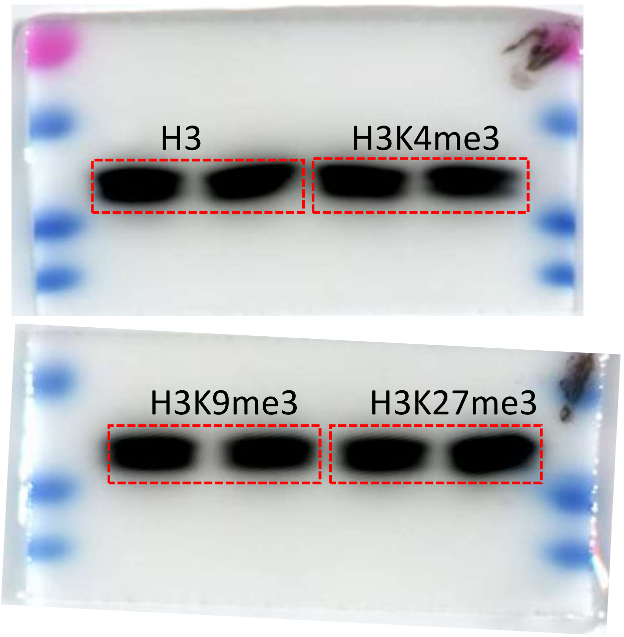

Figure 3I

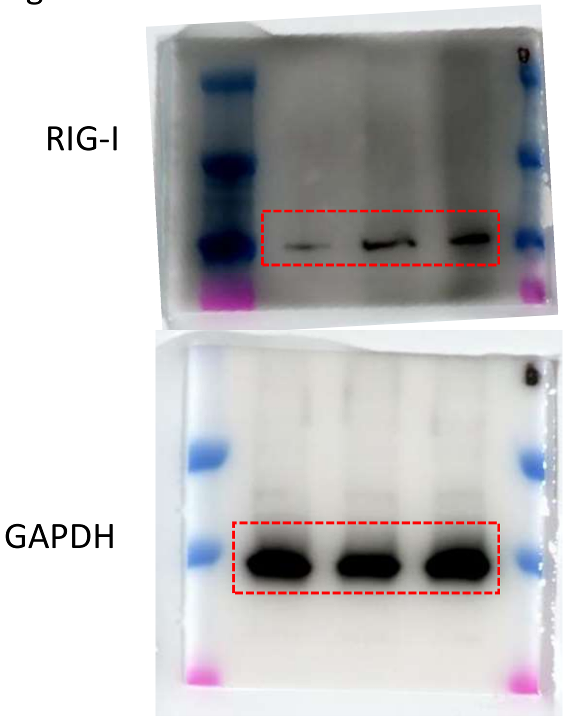

Figure 3B

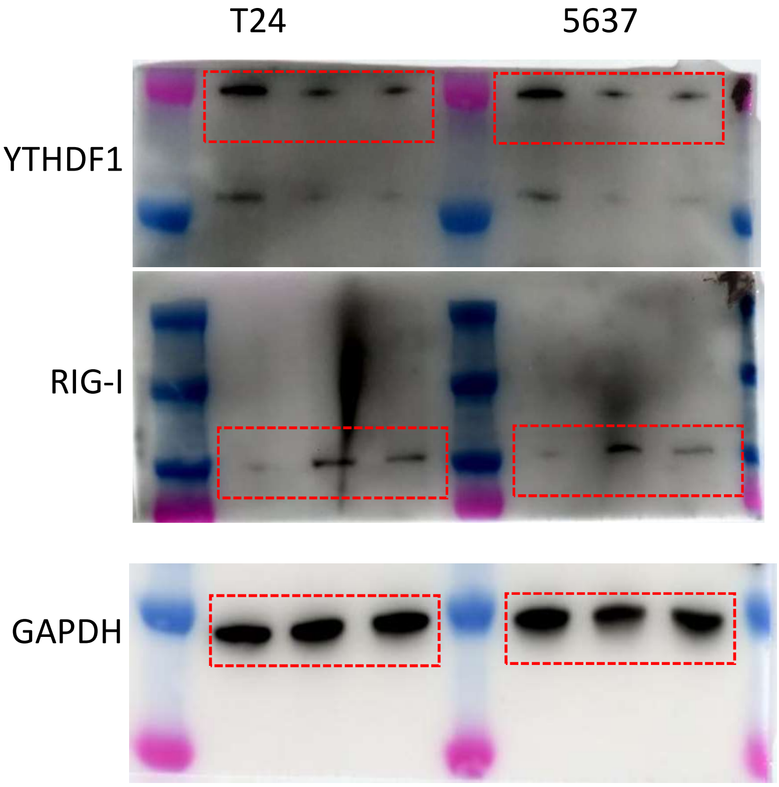

Figure 3M

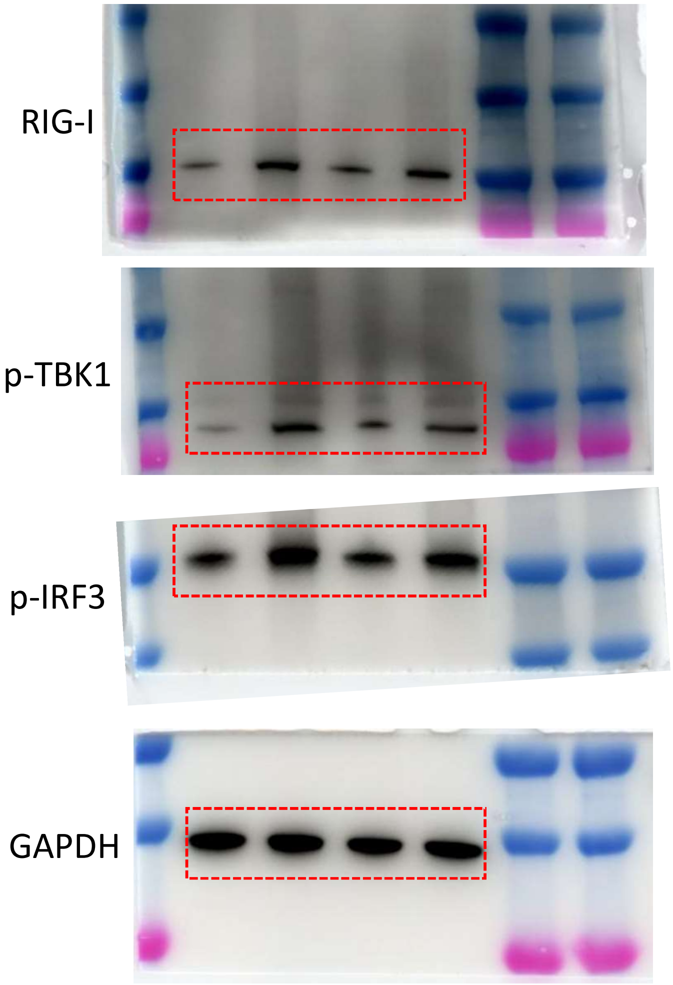

Figure 7D

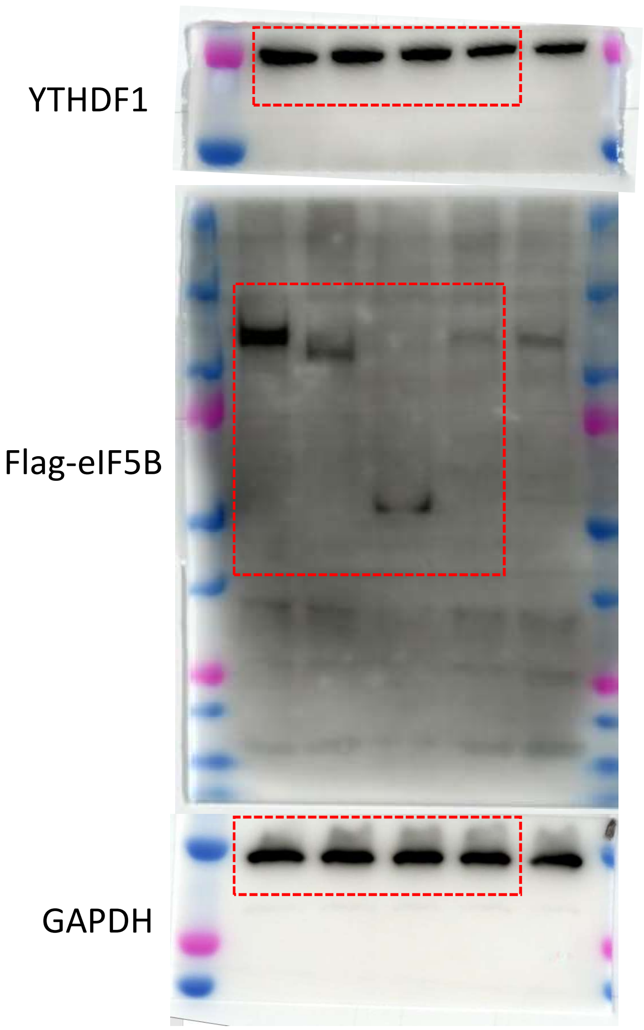

Figure 7E

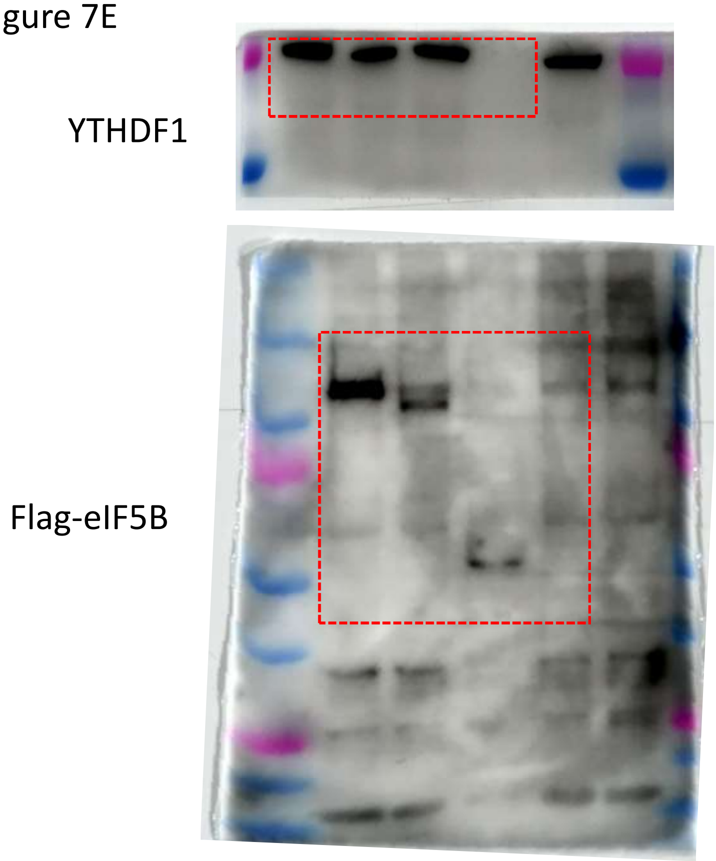

Figure 7J

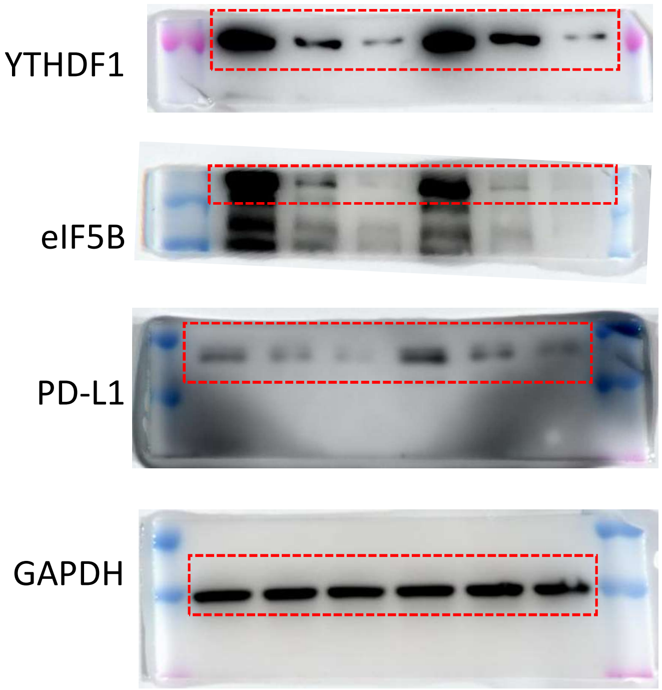

Figure 7K

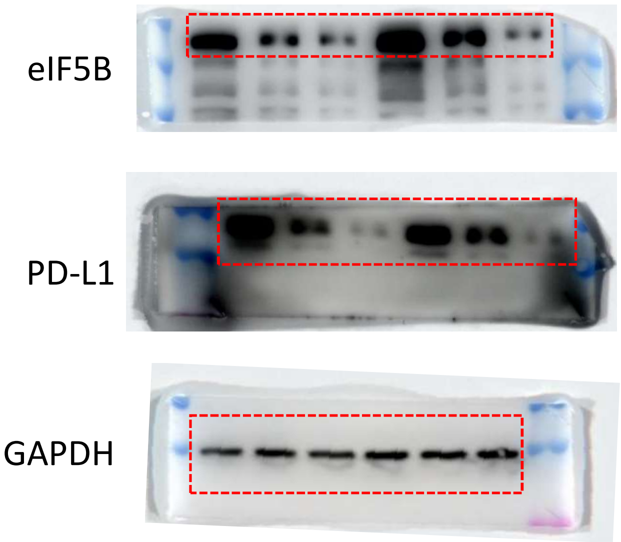

Figure 7L, M

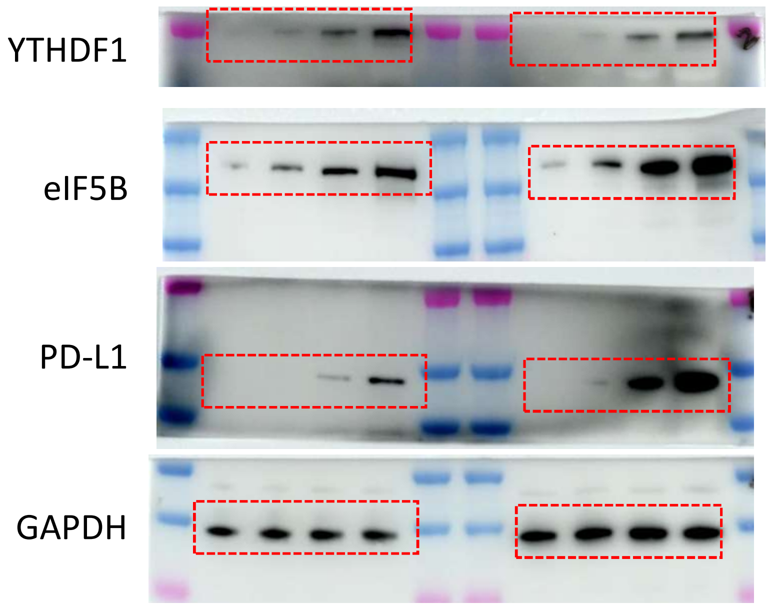

Figure S3C

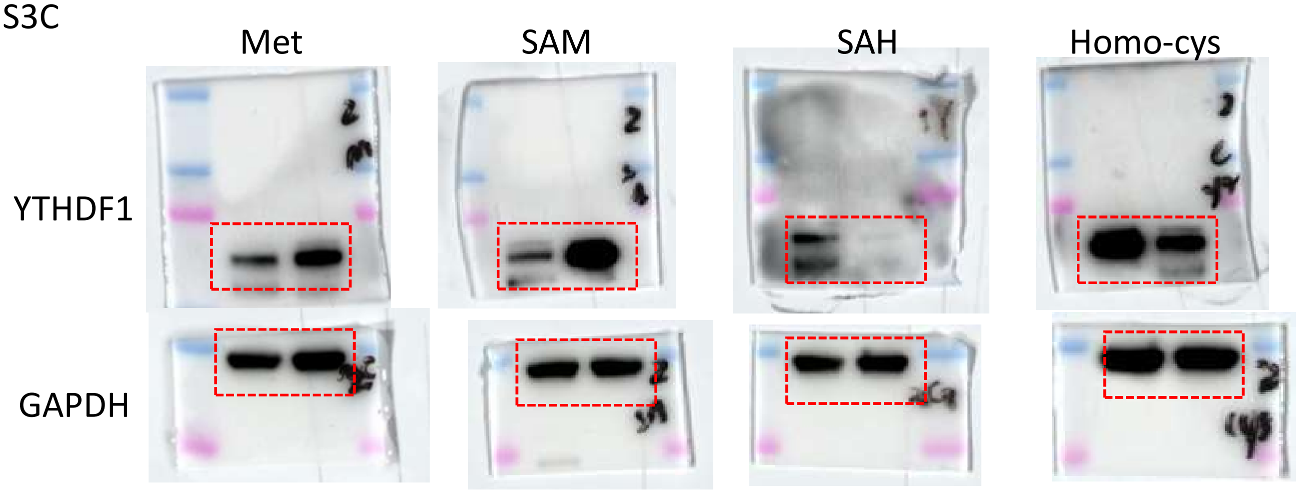

Figure S3D

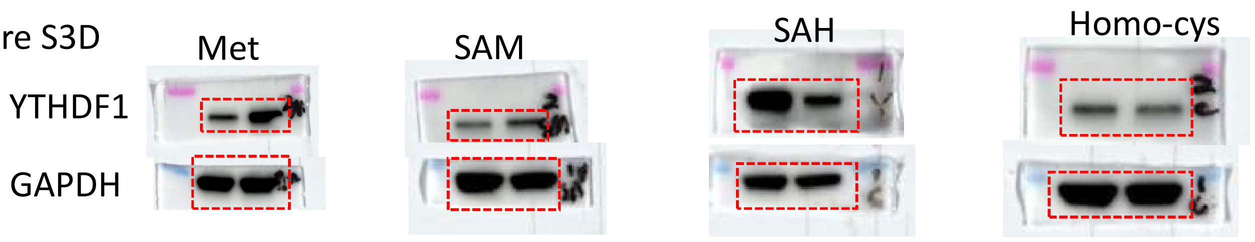

Figure S8B

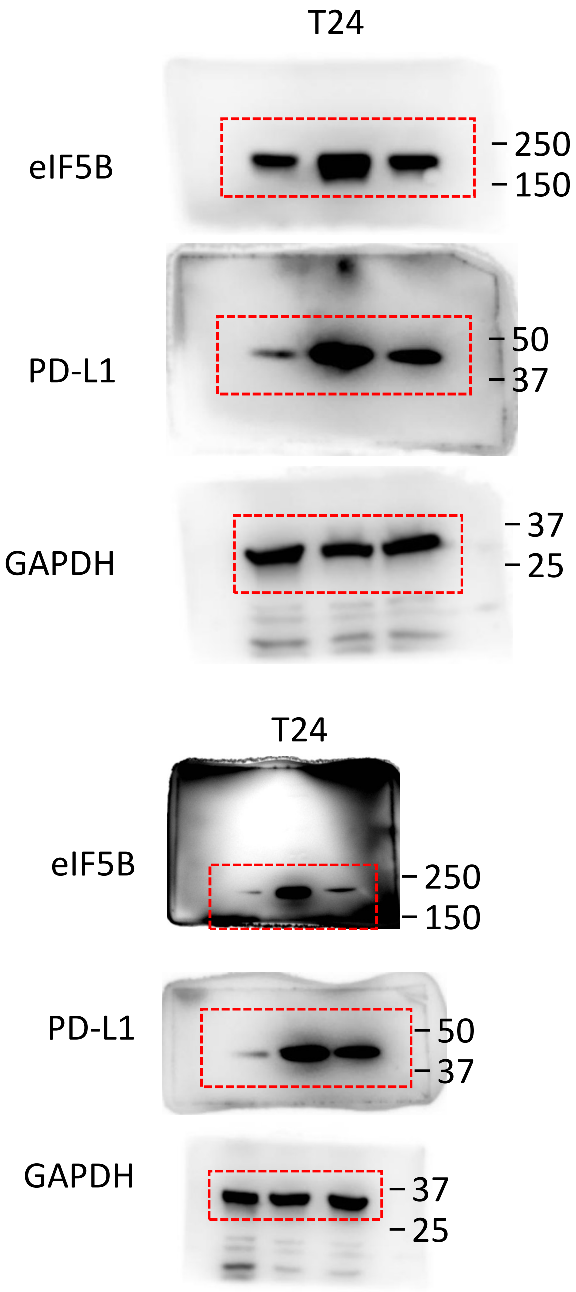

Figure S8C

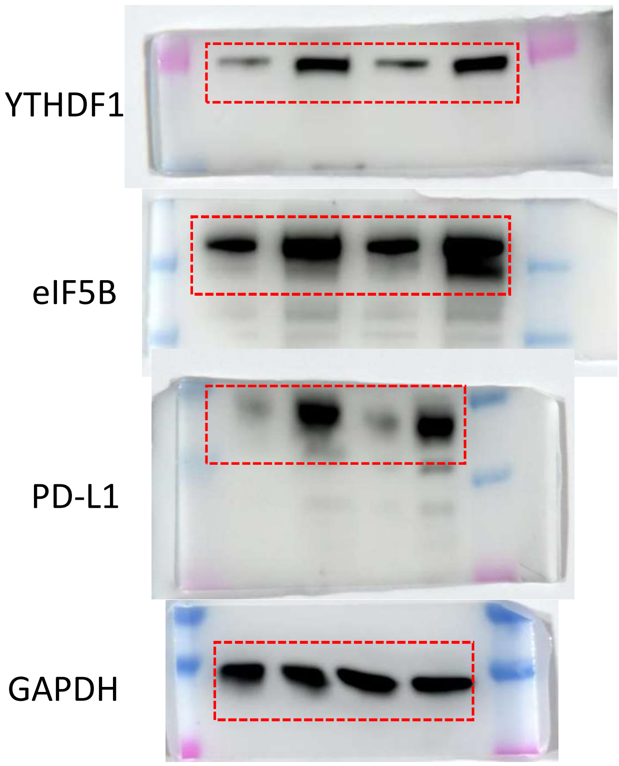

Figure S8D

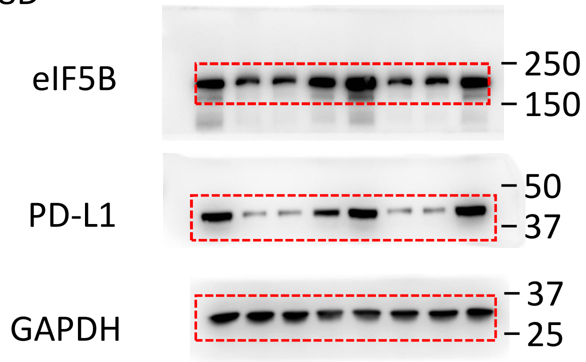

Figure S8E

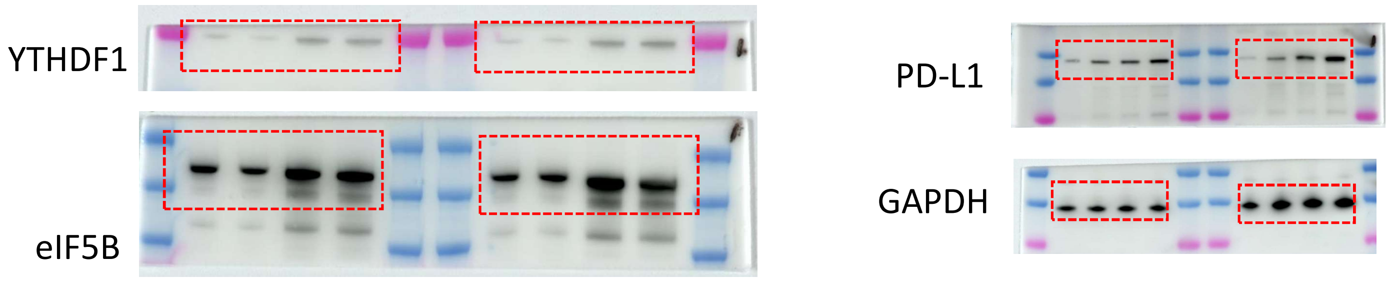

Supplement: Supplementary file 5 — Original WB [file 41418_2024_1434_MOESM5_ESM.pdf]
